# The Structural Effect of FLT3 Mutations at 835th Position and Their Interaction with Acute Myeloid Leukemia Inhibitors: In Silico Approach

**DOI:** 10.3390/ijms22147602

**Published:** 2021-07-16

**Authors:** Abeer M. Al-Subaie, Balu Kamaraj

**Affiliations:** 1 Department of Clinical Laboratory Sciences, College of Applied Medical Sciences, Imam Abdulrahman Bin Faisal University, Dammam 31441, Saudi Arabia; amnalsubaie@iau.edu.sa; 2 Department of Neuroscience Technology, College of Applied Medical Sciences in Jubail, Imam Abdulrahman Bin Faisal University, Jubail 35816, Saudi Arabia

**Keywords:** FLT3, mutations, AML, leukemia, flexibility, interaction, inhibitors

## Abstract

FMS-like tyrosine kinase 3 (FLT3) gene mutations have been found in more than one-third of Acute Myeloid Leukemia (AML) cases. The most common point mutation in FLT3 occurs at the 835th residue (D835A/E/F/G/H/I/N/V/Y), in the activation loop region. The D835 residue is critical in maintaining FLT3 inactive conformation; these mutations might influence the interaction with clinically approved AML inhibitors used to treat the AML. The molecular mechanism of each of these mutations and their interactions with AML inhibitors at the atomic level is still unknown. In this manuscript, we have investigated the structural consequence of native and mutant FLT-3 proteins and their molecular mechanisms at the atomic level, using molecular dynamics simulations (MDS). In addition, we use the molecular docking method to investigate the binding pattern between the FLT-3 protein and AML inhibitors upon mutations. This study apparently elucidates that, due to mutations in the D835, the FLT-3 structure loses its conformation and becomes more flexible compared to the native FLT3 protein. These structural changes are suggested to contribute to the relapse and resistance responses to AML inhibitors. Identifying the effects of FLT3 at the molecular level will aid in developing a personalized therapeutic strategy for treating patients with FLT-3-associated AML.

## 1. Introduction

Acute myeloid leukemia (AML) is characterized by an abnormally high number of blasts in the blood and/or bone marrow (BM). It refers to a category of diseases that have various driver events and pathogeneses and may occur at different stages of the hematopoietic hierarchy [1]. AML genomic studies have shown that many genes are often mutated, resulting in new genomic classifications, predictive biomarkers, and therapeutic targets [2]. In most acute leukemias, the FMS-like tyrosine kinase 3 (FLT3) receptor is overexpressed [3]. FLT3 mutations are typically associated with AML but uncommonly with acute lymphoblastic leukemia (ALL) [4]. The *FLT3* gene is found on chromosome 13q12. It is a member of the receptor tyrosine kinase (RTK) family, which includes KIT, FMS, and PDGFR, among other receptors involved in hematopoiesis regulation [5,6].

*FLT3* gene mutations are found in roughly 30% of all AML cases [7,8]. It has been found to be mutated in more than one-third of AML cases, making it one of the most common molecular genetic alterations and thus a promising therapeutic target [6]. Internal tandem duplication (ITD) mutations of the FLT3 juxtamembrane domain, which are gain-of-function mutations observed in 25–35 percent of newly diagnosed AML patients [6], and tyrosine kinase domain (TKD) point mutations [9] are the two major forms of FLT3 mutations found in newly diagnosed AML patients. The FLT3-ITD and FLT3-TKD mutations trigger FLT3 kinase activity constitutively, resulting in AML proliferation and survival [10]. Multiple intracellular signaling pathways, primarily STAT5, MAPK, and AKT signals, are activated by mutant FLT3, leading to cell proliferation and anti-apoptosis [1,11].

Early detection of FLT3 mutations would aid in a deeper understanding of the patient’s disease and allow for more tailored therapy, which may help patients achieve longer and more stable remissions [2]. Point mutations in the TKD that cause single amino acid substitutions, often in the aspartic acid 835 of the kinase domain, are found in 5–10% of patients [9]. The most common point mutation in FLT3 occurs at the 835th residue (D835A, D835E, D835F, D835G, D835H, D835I, D835N, D835V, and D835Y), frequently in the activation loop region. This aspartic acid has been identified as a critical regulatory residue in the TK receptor and is highly conserved [12,13,14,15]. Although it has long been recognized that the D835 residue is critical in maintaining FLT3 inactive conformation, these mutations are associated with a poor prognosis in patients [16,17,18,19,20].

FLT3 has been identified as a substrate for small-molecule tyrosine kinase inhibitors (TKIs) that are selective and specific [21,22,23,24]. Following treatment with tyrosine kinase inhibitors, mutations involving D835 are the most common genetic mechanism of relapse and resistance in FLT3-mutant AML [25,26,27]. Clinical use of type-I and type-II FLT3 inhibitors has been licensed, resulting in therapeutic paradigms for AML with FLT3 mutations [28]. Ponatinib (AP24534), tandutinib (MLN518), sunitinib (SU11248), sorafenib (DB00398), FF-10101, KW2449, crenolanib (CP-868596), gilteritinib (ASP2215), quizartinib (AC220), and PLX3397 (pexidartinib) are some of the drugs accessible [29,30,31,32,33,34]. An increasing number of TKIs with activity against FLT3 are currently being tested in clinical trials.

Since the discovery of FLT3 mutations in 1996 [12], researchers have eagerly worked to elucidate the molecular mechanisms of normal and abnormal FLT3 signaling pathways. However, while several experimental studies [9,35,36] explain the effect of a point mutation on D835 residue, the structural studies of each of these mutations and their interaction with AML inhibitors are still unknown. The advantage of macromolecule simulation and the integrated computational approaches enables exploration of the biological mechanisms and protein–drug affinity involved in the dysregulated signaling pathways that result in cancer-related diseases [37,38,39]. Hence, in this study, we studied the structural changes of native and mutant (D835A, D835E, D835F, D835G, D835H, D835I, D835N, D835V, and D835Y) FLT3 at the atomic level using the molecular dynamics (MD) simulation approach. Furthermore, we also investigated the binding affinity of AML inhibitors with native and mutant FLT3 proteins by using a molecular docking approach. Our results will aid in the comprehension of the molecular mechanism of D835 mutations in FLT3 protein, and further develop the potential tailored drugs for AML patients.

## 2. Results

### 2.1. Re-Modeling of Native FLT3 Protein and Build the Mutant Structures

Observing the conformational change of FLT3 protein upon mutations and its interaction with AML inhibitors is essential for predicting the 3D conformation of the cytoplasmic domain of FLT3 protein without missing residues. Therefore, we have used the I-TASSER server to re-build the 3D structure of FLT3 protein. The PDB ID: 1RJB was used to re-model the FLT3 protein, and it has shown 87% coverage and similarity with FLT3 cytoplasmic domain protein sequences. Based on a high c-score we have selected the modeled FLT3 protein. Furthermore, the SWISS-PDB tool was applied to build the mutant (D835A, D835E, D835F, D835G, D835H, D835I, D835N, D835V, and D835Y) FLT3 structures. PROCHECK and PROSA online programs were used to assess the quality of predicted model structures of native and mutant FLT3 proteins. The native FLT3-protein showed that 100% favored and allowed region and z-score value of −8.42. Mutant structures showed in the range of 99.1–100% favored and allowed region and z-score values in the range of −8.28 to −8.39. These scores validate the high confidence level for the predicted native and mutants modeled structures and used for further analysis.

### 2.2. MD Simulations

The molecular dynamics simulation (MDS) approach further motivated us to examine the structural behavior of native and mutant (D835A, D835E, D835F, D835G, D835H, D835I, D835N, D835V, and D835Y) FLT3 proteins at the atomic level. We analyzed the root-mean-square deviation (RMSD), root-mean-square fluctuation (RMSF), radius of Gyration (Rg), solvent-accessible surface area (SASA), hydrogen bond (H-bonds), density plot, and dynamic cross-correlation matrix (DCCM) analysis to investigate the differences in structural and functional variations between the native and mutant FLT3 proteins.

#### 2.2.1. RMSD and RMSF Analysis

**Figure 1:**
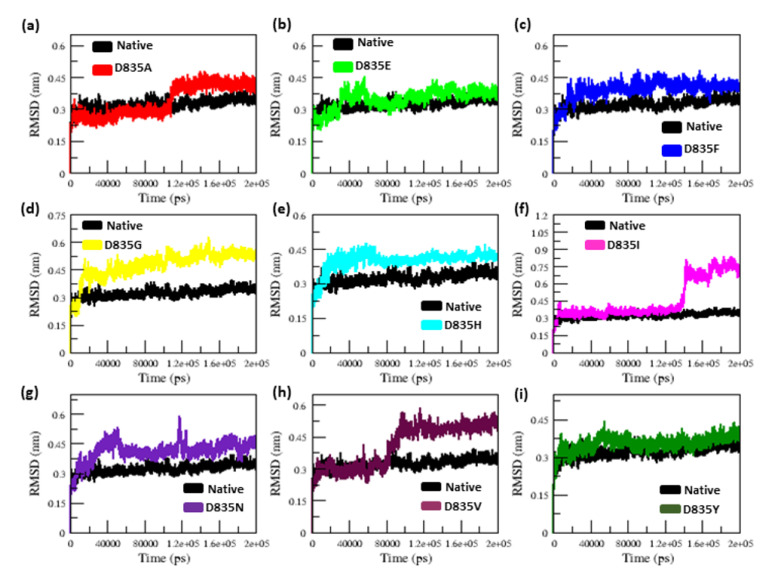
Compared analysis of native and mutants FLT3 backbone RMSD for the period of 200 ns. (**a**) Native vs. D835A; (**b**) Native vs. D835E; (**c**) Native vs. D835F; (**d**) Native vs. D835G; (**e**) Native vs. D835H; (**f**) Native vs. D835I; (**g**) Native vs. D835N; (**h**) Native vs. D835V; (**i**) Native vs. D835Y.

To investigate the convergence of the native and mutant (D835A, D835E, D835F, D835G, D835H, D835I, D835N, D835V, and D835Y) protein system, the RMSD for all Cα-atoms from the initial structure was measured. The native and D835A mutant structures display a similar pattern of deviation up to ~10 ns in the RMSD plot, after which the mutant (D835A) structure shows a progressive rise in RMSD value in comparison to the native structure until the simulation ends (200 ns) (Figure 1a).

The D835E mutant structure shows a similar way of deviation as the native structure from starting up until ~34 ns, but then shows an increase in the RMSD value until the end of the simulation, as illustrated in Figure 1b. The D835F mutant structure exhibited a similar deviation from the beginning to ~30 ns. Later, the D835F mutant showed more deviation compared to the native structure. (Figure 1c). The D835G and D835H mutant structures attained the maximum deviation from the beginning (0 ns) to end (200 ns) of the simulation compared to the native structure, as illustrated in Figure 1d,e, respectively. The D835I mutant structure shows a similar way of deviation from the beginning up to ~130 ns, then the mutant (D835I) attained a rise in RMSD value (Figure 1f) compared to the native structure till the end of the simulation. The D835N mutant, from the beginning to ~25 ns, shows a similar way of deviation after it shows an increase rise in the deviation than the native structure (Figure 1g). On the other hand, the D835V and D835Y mutants exhibit a similar deviation from starting until ~82 ns and ~48 ns, respectively, after both mutants attained maximum deviations compared to the native structure, shown in Figure 1h,i, respectively. The average RMSD value of the native and mutant (D835A, D835E, D835F, D835G, D835H, D835I, D835N, D835V, and D835Y) structures are listed in Table 1.

**Figure 2:**
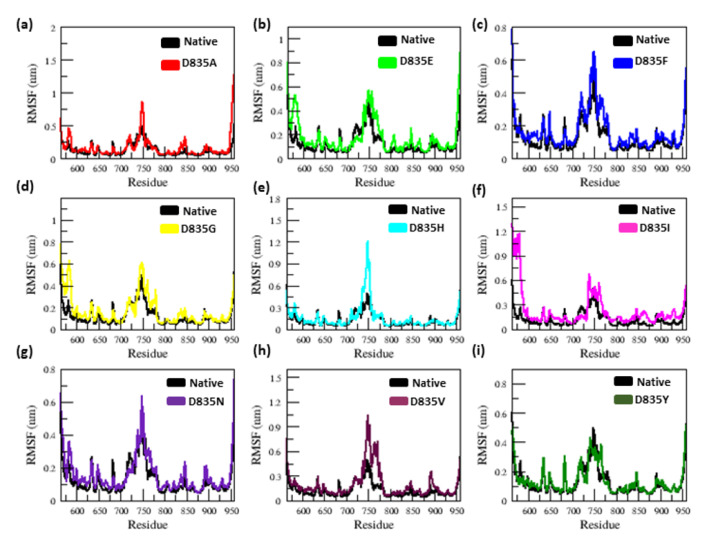
The C-α RMSF simulation data for native and mutant FLT3 protein structure’s residues during the time span of 200 ns. (**a**) Native vs. D835A; (**b**) Native vs. D835E; (**c**) Native vs. D835F; (**d**) Native vs. D835G; (**e**) Native vs. D835H; (**f**) Native vs. D835I; (**g**) Native vs. D835N; (**h**) Native vs. D835V; (**i**) Native vs. D835Y.

The RMSF value analysis revealed a large difference in the fluctuation of residues between the native and mutant (D835A, D835E, D835F, D835G, D835H, D835I, D835N, D835V, and D835Y) FLT3 structures (Figure 2a–i).

The average RMSF value of native and mutant FLT3 proteins is again listed in Table 1. The mutant D835A, D835E, D835F, D835G, D835H, D835I, D835N, and D835V structures have a greater degree of fluctuation in the residue of 835 and neighboring residues than native FLT3 throughout the simulation; this is shown in Figure 2a–h, respectively. Whereas, the D835I mutant shows a higher degree of fluctuation in the residue of 835 and similar fluctuation in the neighboring resides than the native structure (Figure 2i).

#### 2.2.2. Secondary Structure Analysis of Native and Mutant FLT3 Proteins

Through DSSP analysis, we observed how the D835 mutations could disturb the secondary structural conformation of FLT3 protein. We calculated the percentage variation of secondary structure elements of native and mutants FLT3, as listed in Table 2. Helix, β-sheets, coils, and turns were observed in both native and mutant FLT3 proteins. The native structure shows more helix content compared to other mutant structures (Table 2), whereas the coil and turns show more percentages in all mutant structures compared to native FLT3 protein (Table 2).

#### 2.2.3. Geometry and Surface Analysis of Native and Mutants FLT3 Protein

**Figure 3:**
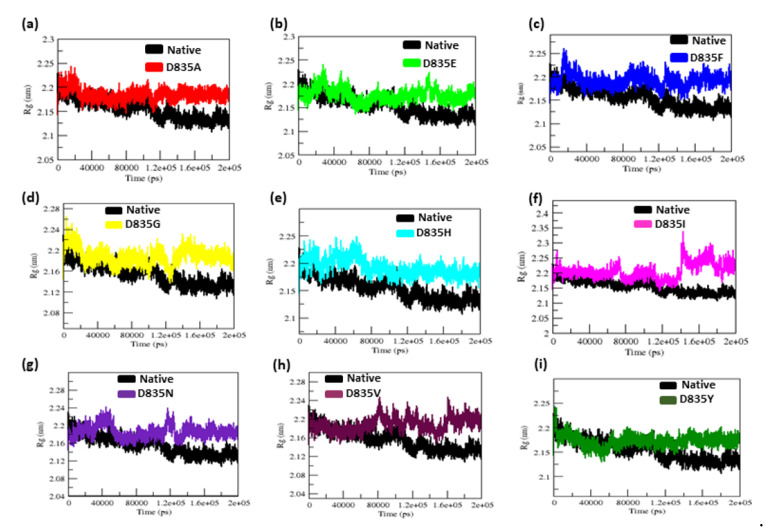
The native and mutant FLT3 protein structures compactness analysis by Radius of gyration for the period of 200 ns. (**a**) Native vs. D835A; (**b**) Native vs. D835E; (**c**) Native vs. D835F; (**d**) Native vs. D835G; (**e**) Native vs. D835H; (**f**) Native vs. D835I; (**g**) Native vs. D835N; (**h**) Native vs. D835V; (**i**) Native vs. D835Y.

By analyzing the radius of gyration (Rg) and solvent-accessible surface area (SASA) plots, we were able to determine the geometry and surface of native and mutant (D835A, D835E, D835F, D835G, D835H, D835I, D835N, D835V, and D835Y) FLT3 proteins. The radius of gyration (Rg) in the native and mutant FLT3 protein structures suggests a degree of compactness. Figure 3a–i shows the Rg plot for Cα atoms in native and mutant FLT3 proteins over time at 300 K. The average Rg values of the native and mutant (D835A, D835E, D835F, D835G, D835H, D835I, D835N, D835V, and D835Y) FLT3 proteins are again listed in Table 1.

In Figure 3a,b, the D835A and D835E mutant structures show similar Rg values from the beginning to ~120 ns, after which it rises and shows more Rg value than the native structure until the end (200 ns) of the simulation. The D835F mutant shows more Rg value than the native structure from the ~15 ns, to the end of the simulation (Figure 3c). The D835G, D835H, and D835I mutants show more Rg value than the native structures from the beginning to end of the simulation and shown in Figure 3d–f, respectively. Mutant D835N shows a similar Rg value from the beginning to ~35 ns, after it shows more Rg value than the native structures until the end of the simulation (Figure 3g). In Figure 3h, from the beginning to ~75 ns, the Rg value of the D835V mutant is similar to the native structure, but after that, the Rg value of the D835V mutant is higher than the native structure until the end of the simulation. On the other hand, the D835Y shows a lesser Rg value than the native structure from starting to ~63 ns after it rises, and shows more Rg value than the native structure until the end of the simulation (Figure 3i).

**Figure 4:**
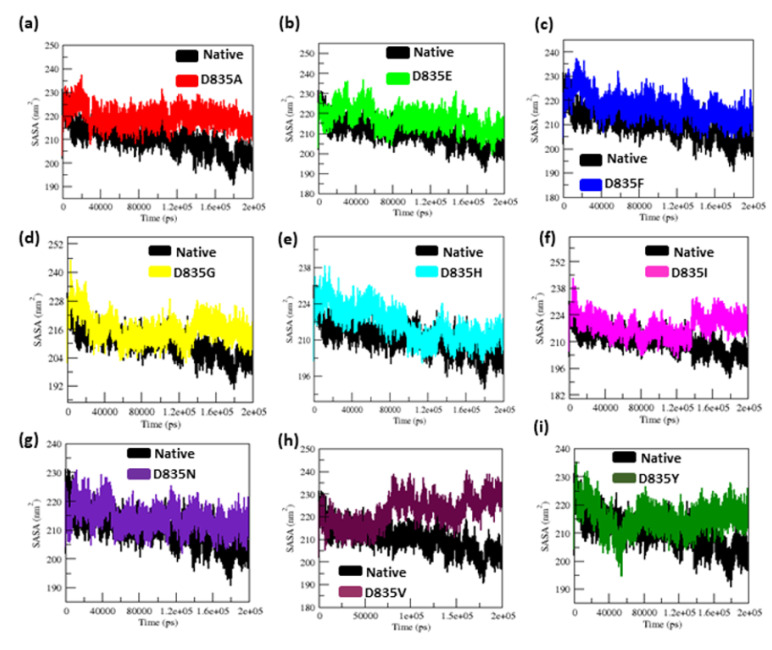
The solvent-accessible surface area (SASA) analysis for native and mutant FLT3 protein structures for the time frame of 200 ns. (**a**) Native vs. D835A; (**b**) Native vs. D835E; (**c**) Native vs. D835F; (**d**) Native vs. D835G; (**e**) Native vs. D835H; (**f**) Native vs. D835I; (**g**) Native vs. D835N; (**h**) Native vs. D835V; (**i**) Native vs. D835Y.

The changes of solvent-accessible surface area (SASA) of native and mutants (D835A, D835E, D835F, D835G, D835H, D835I, D835N, D835V, and D835Y) FLT3 protein over time are depicted in Figure 4a–i.

The average SASA value of native and mutant FLT proteins is again listed in Table 1. In Figure 4a–f, from the beginning to the end of the simulation, the D835A, D835E, D835F, D835G, D835H, and D835I mutants have higher SASA values than the native structure. The mutant D835N, shows a similar SASA value to the native structure from the beginning to ~3.4 ns, after which it rises and shows more SASA value than the native structure till the end of the simulation (Figure 4g). The mutant D835V, shows a similar SASA value from the starting to ~75 ns, after which it increases and shows more SASA value than the native structure till the end of the simulation as shown in Figure 4h. The D835Y shows more SASA value than the native structure from the 150 ns to the end of the simulation and shown in Figure 4i.

#### 2.2.4. NH-Bonds, Density and DCCM Plot Analysis

**Figure 5:**
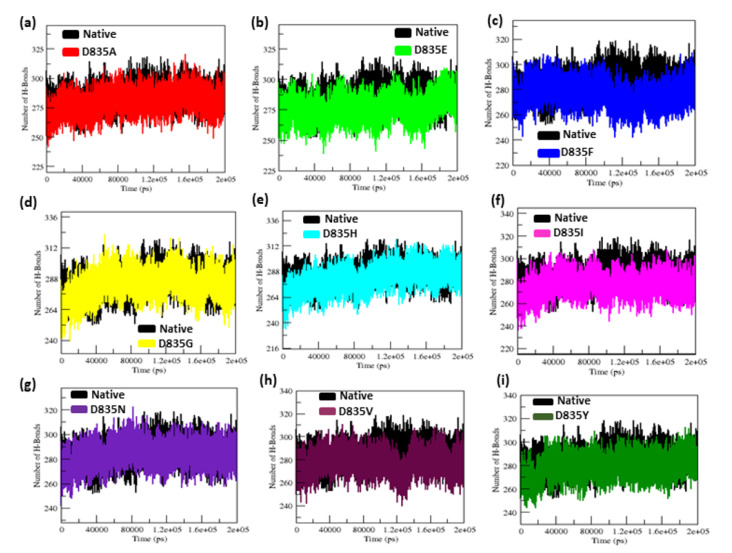
Intramolecular H-bond simulation data for native and mutant FLT3 protein structures for 200 ns, (**a**–**i**) elucidating a clear difference in H-bonds between native and mutant FLT3 structures. (**a**) Native vs. D835A; (**b**) Native vs. D835E; (**c**) Native vs. D835F; (**d**) Native vs. D835G; (**e**) Native vs. D835H; (**f**) Native vs. D835I; (**g**) Native vs. D835N; (**h**) Native vs. D835V; (**i**) Native vs. D835Y.

The protein folding, stability, and function are all dependent on the hydrogen bond. To better understand the stability of native and mutant (D835A, D835E, D835F, D835G, D835H, D835I, D835N, D835V, and D835Y) FLT3 proteins, we measured the intramolecular H-bond with respect to time (Figure 5a–i).

**Figure 6:**
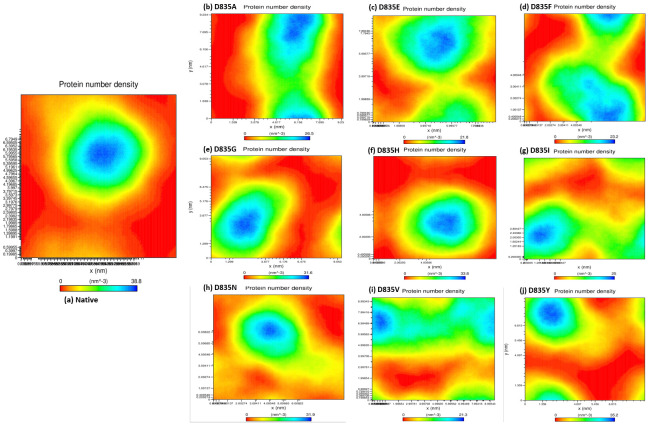
The atomic density distribution of native and mutant FLT3 structures for the period of 200 ns. (**a**) Native; (**b**) D835A; (**c**) D835E; (**d**)D835F; (**e**)D835G; (**f**)D835H; (**g**)D835I; (**h**)D835N; (**i**)D835V; (**j**) D835Y.

The average number of hydrogen bonds of native and mutant FLT3 proteins are 284.85 ± 9.20, 279.91 ± 9.17, 276.11 ± 9.09, 276.58 ± 8.99, 283.47 ± 9.95, 281.06 ± 11.23, 275.78 ± 9.15, 283.65 ± 8.96, 277.54 ± 9.04, and 279.04 ± 8.85 (Table 1), respectively. Furthermore, the atomic density distribution plot clearly demonstrated the results of molecular changes in the protein upon mutations. Figure 6a–j shows that the atomic density distribution of the native and mutant structures (D835A, D835E, D835F, D835G, D835H, D835I, D835N, D835V, and D835Y) is 38.8 nm^3^, 26.5 nm^3^, 21.8 nm^3^, 23.2 nm^3^, 31.6 nm^3^, 33.8 nm^3^, 25 nm^3^, 31.9 nm^3^, 21.3 nm^3^, and 35.2 nm^3^, respectively.

**Figure 7:**
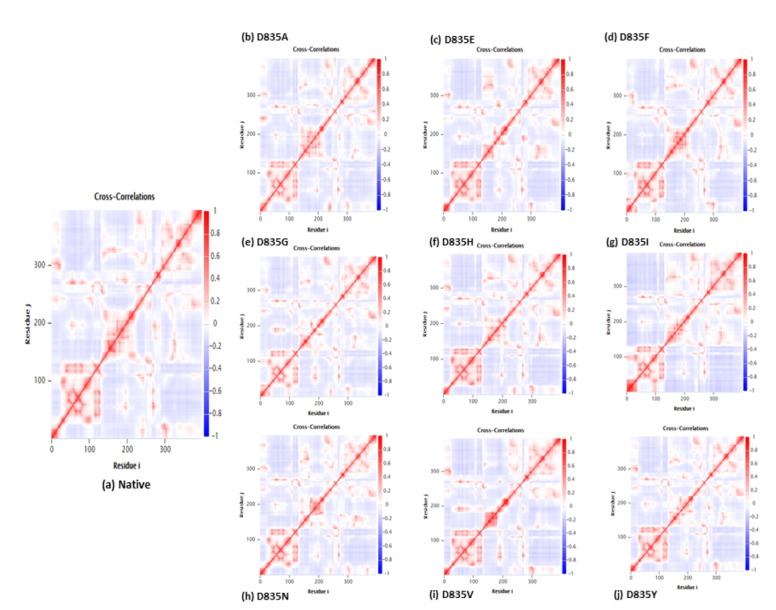
Native and mutant FLT3 proteins cross-correlation matrix of the fluctuations of coordinates for Cα atoms around their mean positions during MD simulation; the extent of correlated motion (red color) and anti-correlated motions (blue color) are color-coded. (**a**) Native; (**b**) D835A; (**c**) D835E; (d)D835F; (**e**)D835G; (**f**)D835H; (**g**)D835I; (**h**)D835N; (**i**)D835V; (**j**) D835Y.

We investigated the correlation between the motions of residues of native and mutant FLT3 trajectories by creating the DCCM matrix. The C(i,j) components of the cross-correlation matrix are symmetrical around the diagonal. Since these maps have been normalized, the magnitude of the correlation can be calculated by measuring the cross-correlation coefficient between the atomic displacements. The strongly positive regions appeared in red color, which shows a strong correlation in the motions of residues, while the negative regions appeared in dark blue, which shows a strong anti-correlated motion of the residues. The better two atoms are correlated, based on the higher cross-correlation value (Figure 7a–j).

**Figure 8:**
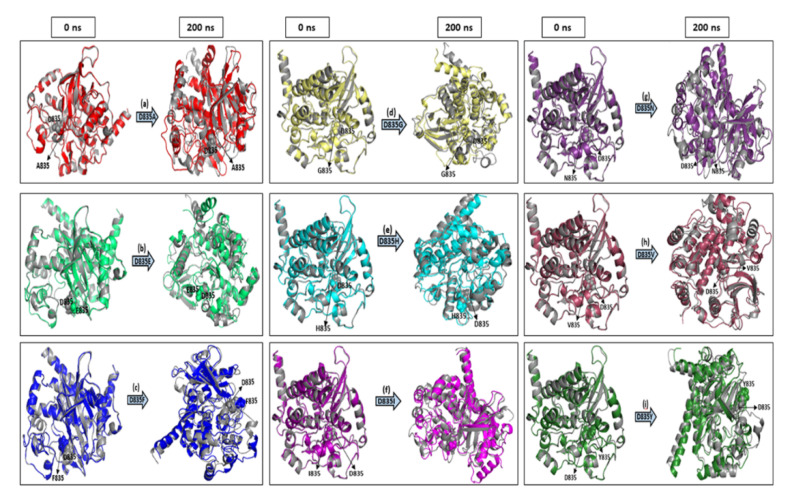
The beginning (0 ns) and end (200 ns) of the simulation snapshots of native and mutant FLT3 protein conformation were superimposed and represented in cartoon style. (**a**) Native vs. D835A (D835A mutant displayed in red color); (**b**) Native vs. D835E (D835A mutant displayed in green color); (**c**) Native vs. D835F (D835F mutant displayed in blue color; (**d**) Native vs. D835G (D835G mutant displayed in yellow color); (**e**) Native vs. D835H (D835H mutant displayed in cyan color); (**f**) Native vs. D835I (D835I mutant displayed in magenta color); (**g**) Native vs. D835N (D835N mutant displayed in purple color); (**h**) Native vs. D835V (D835V mutant displayed in maroon color); (**i**) Native vs. D835Y (D835Y mutant displayed in dark green color). Native FLT3 in all the snapshots shown in grey color. The mutation residues are displayed in sphere model.

To observe the structural changes, we have shown in Figure 8, the beginning (0 ns) and end (200 ns) of the timescale of native and mutant FLT3 proteins.

### 2.3. Docking Analysis of AML Inhibitors with Native and Mutants FLT3 Proteins

Further, we investigated the binding behavior of clinically approved type-I and type-II AML inhibitors with native and mutant (D835A, D835E, D835F, D835G, D835H, D835I, D835N, D835V, and D835Y) FLT3 by using a molecular docking approach.

In this study, we docked the MD output (average structure at 200 ns) of the native and mutant FLT3 proteins with 10 AML inhibitors (Crenolanib, FF-10101, Gilteritinib, KW-2449, PLX3397, Ponatinib, Quizartinib, Sorafenib, Sunitinib, and Tandutinib) to observe the interaction behavior of individual D835 mutations of FLT3 proteins with inhibitors. The two-dimensional structure of all AML inhibitors is depicted in Figure S1. Based on a literature review, the active site residues GLU-661, GLU-692, CYS-694, CYS-695, ASP-829, and ARG-834 were considered [40,41,42,43]. The binding energy of native and mutant FLT3 proteins with AML inhibitors listed in Table 3.

The average binding affinity between the native FLT3 protein and crenolanib inhibitor was found to be −8.9 kcal/mol. Whereas, the average binding energies of mutant (D835A, D835E, D835F, D835G, D835H, D835I, D835N, D835V, and D835Y) FLT3 proteins and the crenolanib inhibitor are −8.5, −10.4, −9.7, −10.2, −9.2, −9.0, −9.6, −10.4, and −8.9 kcal/mol, respectively (Table S1). The FF-10101 inhibitor has an average binding energy with the native and mutant FLT3 proteins of −8.3, −5.8, −7.7, −7.8, −5.9, −7.6, −8.6, −8.2, −8.8, and −8.3 kcal/mol, respectively (Table S2). The gilteritinib inhibitor has average binding energies of −8.5, −6.7, −7.3, −8.1, −7.6, −9.3, −8.0, −4.8, −9.6, and −8.9 kcal/mol, with native and mutant FLT3 proteins, respectively (Table S3). The average binding energies of the KW-2449 inhibitor with native and mutant FLT3 are −9.9, −8.8, −10.7, −9.2, −9.9, −8.3, −8.7, −9.5, −10.6, and −9.5 kcal/mol, respectively (Table S4). The PLX3397 inhibitor has an average binding energy with native and mutant FLT3 proteins of −9.7, −9.6, −10.1, −9.2, −9.9, −9.0, −9.9, −9.8, −10.1, and −10.1 kcal/mol, respectively (Table S5).

Ponatinib inhibitor shows an average binding energy with native and mutant FLT3 proteins are −10.3, −7.5, −7.9, −8.4, −8.4, −10.1, −10.0, −7.7, −12.2, and −9.9 kcal/mol, respectively (Table S6). Whereas, the Quizartinib inhibitor has an average binding energy with native and mutant FLT3 proteins of −9.5, −7.3, −6.4, −8.4, −3.8, −9.5, −8.2, −5.0, −10.5, and −11.1 kcal/mol, respectively, and listed in Table S7. The average binding energy between the Sorafenib inhibitor and native and mutant FLT3 proteins are −10.4, −8.3, −8.3, −9.2, −10.5, −9.5, −10.8, −9.7, −10.8, and −10.4 kcal/mol, respectively (Table S8). Sunitinib inhibitor has an average biding energy with native and mutant FLT3 proteins of −8.3, −6.4, −9.3, −8.1, −9.4, −8.0, −8.4, −8.3, −9.0, and −8.7 kcal/mol, respectively, and listed in Table S9. The tandutinib inhibitor shows the average binding energy with native and mutant FLT3 proteins are −9.6, −7.1, −7.5, −7.4, −7.2, −8.7, −9.8, −8.6, −9.9, and −9.3 kcal/mol, respectively (Table S10).

The number of hydrogen bonds, and interactive residues of AML inhibitors (Crenolanib, FF-10101, Gilteritinib, KW-2449, PLX3397, Ponatinib, Quizartinib, Sorafenib, Sunitinib, and Tandutinib) with native and mutant (D835A, D835E, D835F, D835G, D835H, D835I, D835N, D835V, and D835Y) FLT3 proteins are depicted in Tables S1–S10, respectively. Additionally, the interacting residues of the native and mutant FLT3 proteins with AML inhibitors were displayed in Figures S2–S11.

## 3. Discussions

The early recognition of FLT3 mutations would help aid in a deeper understanding of the patient’s ailment and permit further personalized therapy, which could support patients in attaining extended and more stable remissions. Scientists in the last two decades have been determined to clearly understand the molecular mechanisms of regular and irregular FLT3 signaling pathways. Several experimental studies [9,35,36] elucidate the consequence of a point mutation on D835 residue of FLT3, but the structural study of these mutations and their interactional behavior with AML inhibitors is still unknown. Our research focused on elucidating the structural consequences of FLT3 protein upon point mutations on D835. We have used an integrated computational approach to explore structural transition which is imposed by nine mutations, namely, D835A, D835E, D835F, D835G, D835H, D835I, D835N, D835V, and D835Y, on the native FLT3 structure and its interaction with type-I and type-II AML inhibitors. Predicting the three-dimensional conformation of the cytoplasmic domain of FLT3 protein without mislaid residues is important to observe the structural variation on FLT3 protein upon mutations and its interaction with AML inhibitors. Thus, we applied the I-TASSER server to re-build the three-dimensional structure of FLT3 protein and used it as input for MDS and docking studies. MDS was performed using several parameters such as RMSD, RMSF, Rg, SASA and NH-bond, Density Plot, and covariance matrix to evaluate the stability and dynamic behavior of native and mutant FLT3 proteins. The above MD parameters average values of native and mutant FLT3 proteins were shown in Table 1.

The RMSD plots show the convergence for the native and mutants FLT3 system throughout the simulation and producing the stable conformation, thus providing a suitable basis for further analysis. Furthermore, in the RMSD plot, mutant (D835A, D835E, D835F, D835G, D835H, D835I, D835N, D835V, and D835Y) structures exhibited higher deviation, whereas native structure showed lower deviation (Figure 1a–i). It designates that the D835 mutations have severe changes in the conformational geometry of FLT3 protein. Through the aim of determining the percentage of secondary structural elements, we observed how the mutations at the D835 position could disturb the secondary structural conformation of native FLT3 protein. The percentage of the secondary structural elements of the native and mutant FLT3 proteins was displayed in Table 2. It clearly explained that due to mutations (D835A, D835E, D835F, D835G, D835H, D835I, D835N, D835V, and D835Y) it undergoes major structural changes in the protein conformation. It was that less helical content was observed in all mutant structures, but more coil and turns content was observed in all the mutant structures compared to native FLT3 protein. In general, the helix was more stable and rigid in nature, whereas coils and turns are more flexible in nature in protein conformation [44]. Hence, it confirmed that due to mutation, the native structure lost the rigidity and became more flexible, which, in turn, affects the structure conformation and function of FLT3 protein. It was also well supported by Rg, SASA, RMSF, and NH bond analysis.

The radius of gyration is a crucial constraint that predicts the compactness of proteins in solution [45,46,47,48]. SASA is a good measure of structure geometry of protein [46,47,48,49,50]. With the above existing phenomena, the Rg, and SASA plots showed significant outcomes. Our Rg analysis showed major fluctuation in mutant (D835A, D835E, D835F, D835G, D835H, D835I, D835N, D835V, and D835Y) structures as compared to the native FLT3 proteins, till the end of the simulation as shown in Figure 3a–i. The variation in SASA of the native and mutant FLT3 structures with time is shown in Figure 4a–i. Mutant (D835A, D835E, D835F, D835G, D835H, D835I, D835N, D835V, and D835Y) FLT3 structures showed higher values of SASA with time whereas native structure showed lower values of SASA with time. Furthermore, we determined the residues of FLT3 protein flexibility upon mutations via RMSF analysis. Toward the end of the simulation, greater flexibility was observed in mutant structures compared to native FLT3 protein (Figure 2a–i). It further confirms that mutant protein residues were flexible throughout the simulation and it is attained the extended conformation as compared to native FLT3 protein (Figure 2a–i). Hence, the mutant (D835A, D835E, D835F, D835G, D835H, D835I, D835N, D835V, and D835Y) FLT3 structures showed higher fluctuation in RMSD, secondary structural elements, Rg, SASA, and RMSF plot, which specifies that the native FLT3 protein might be enduring a major structural change and interrupts the stability and the functional behavior of the protein.

The interaction pattern of the corresponding protein is altered when amino acid residues are changed, especially the formation of H bonds [49,50]. Formations of hydrogen bonds in protein structure affect its conformational flexibility [51,52,53,54,55]. Hence, the number of hydrogen bonds (NH bond) is calculated in our analysis for native and mutant FLT3 structures during the simulation time. Prominent changes were observed between the native and mutant FLT3 structures as shown in Figure 5a–i. More number of NH bonds were observed in native FLT3 protein and might support retaining its innate conformational orientation. Whereas, the mutant (D835A, D835E, D835F, D835G, D835H, D835I, D835N, D835V, and D835Y) FLT3 structures show a relatively lower number of H-bond formation, which eventually causes greater flexibility in its conformation. For further reinforcement of our results, we analyzed the atomic density plot and DCCM matrix analysis to attain a better judgement on the MD of the native and mutant FLT3 structures. The mutant structures showed the maximum density distribution over the native FLT3 protein (Figure 6a–j). Whereas in the DCCM matrix, the mutant structures have more motion between the atoms compared to native structures attributed to the mutations (Figure 7a–j). To observe the structural changes, all the mutants (D835A, D835E, D835F, D835G, D835H, D835I, D835N, D835V, and D835Y) structures were superimposed with native confirmation at 0 ns and 200ns and displayed in Figure 8a–i. Therefore, MD simulation results confirm that mutant (D835A, D835E, D835F, D835G, D835H, D835I, D835N, D835V, and D835Y) structures showed conformational transition (changed into flexible nature), which may influence the interaction with AML inhibitors. The MD simulation results clearly designate the consequence of mutations on the 835th residue of FLT3 protein translated into the effect on its structural orientation which might affect its interaction with AML inhibitors.

To further confirm this, we applied a molecular docking method to evaluate the interaction between the MD average structures of native and mutant FLT3 with type-I and type-II AML inhibitors; Crenolanib, FF-10101, Gilteritinib, KW-2449, PLX3397, Ponatinib, Quizartinib, Sorafenib, Sunitinib, and Tandutinib. We used AutoDock Vina 4.2 programs to investigate the interaction between the native and mutant FLT3 proteins and inhibitors. The increased affinity between the receptor and drug molecules was calculated based on the binding energy and number of H-bonds.

In general, the greater and lesser interaction between the protein and drug molecules was considered by the following criteria [56,57,58]. The principle of lower binding energy, along with higher number of H-bonds, shows the greater interactions between the protein and drug molecules, whereas the principle of lower binding energy and lower number of H-bonds, or higher binding energy with high/lower number of H-bonds, represents the lesser interactions between the protein and drug molecules [56,57,58]. With this existing phenomenon, we identified the interaction mechanism of native and mutant FLT3 proteins with AML inhibitors. The binding scores, number of H-bonds, and interactive residues between the native and mutant FLT3 protein with crenolanib inhibitor displayed in Table S1, and Figure S2, respectively.

The mutants D835A, and D835Y show similar/more binding energy and a lower number of H-bonds with crenolanib inhibitor, compared to native FLT3protein. However, the mutants, namely D835F, D835G, D835H, D835N, and D835V, exhibited the least binding energy and a fewer number of H-bonds with crenolanib inhibitor compared to native FLT3 proteins. This clearly indicates that the above mutations lost the interaction and became resistant to the crenolanib inhibitor. The mutants D835E and D835I show the least binding energy and maximum number of H-bonds with crenolanib inhibitors than native and other mutant FLT3 proteins. Hence, these two mutations may become unresistant towards crenolanib inhibitor (Table S1).

The binding scores, number of H-bonds, and interactive residues between the native and mutant FLT3 protein with FF-10101 inhibitor were analyzed and presented in Table S2 and Figure S3, respectively. The mutants D835A, D835E, D835F, D835G, D835H, and D835N lost the interaction and became resistant to the FF-10101 inhibitor, which is attributed to their higher binding energy and smaller number of H-bonds with the FF-10101 inhibitor compared to native FLT3 protein. However, mutants D835I, D835V, and D835Y may become unresistant to the FF-10101 inhibitor, as both exhibit the least interaction and more H-bonds with the FF-10101 inhibitor compared to the native FLT3 protein (Table S2).

On the other hand, the interaction between the gilteritinib inhibitor and both native and mutant FLT3 proteins were analyzed and displayed in Figure S4. The mutants D835H, D835V, and D835Y exhibited the least binding energy and greater number of H-bonds with gilteritinib inhibitor than native and other mutant FLT3 proteins (Table S3). The mutants D835H, D835V, and D835Y may become unresistant towards the gilteritinib inhibitor, whereas other mutants might lose the interaction and may become resistant to the gilteritinib inhibitor (Table S3). The interaction between the native and mutant FLT3 proteins with the KW-2449 inhibitor were also analyzed, as illustrated in Figure S5. The mutants D835E and D83V show lower binding energy and a higher number of H-bonds with KW-2449 inhibitor and may become unresistant to the KW-2449 inhibitor (Table S4). Other mutants (D835A, D835F, D835G, D835H, D835I, D835N, and D835Y) lose the interaction and may become resistant to the KW-2449 inhibitor (Table S4).

The native and mutant FLT3 protein interaction with PLX3397 inhibitor displayed in Figure S6. The mutants D835E, D835G, D835V, and D835Y show lower binding energy and a greater number of H-bonds compared to native and other mutants, and it may become unresistant to the PLX3397 inhibitor. Other mutants (D835A, D835F, D835H, D835I, and D835N) of the FLT3 protein lose the interaction and could become resistant to PLX3397 inhibitors (Table S5). The mutant D835V shows better affinity and higher interaction energy with both ponatinib (Table S6) and tandutinib (Table S10) inhibitors compared to native and other FLT3 mutants. This indicates that D835V mutations could become unresistant to both ponatinib and tandutinib inhibitors, whereas other mutants might have lost the interaction and become resistant towards ponatinib and tandutinib inhibitors. The native and mutant FLT3 protein interactions with ponatinib and tandutinib inhibitors are displayed in Figures S7 and S11, respectively.

The native and mutant FLT3 protein interactions with quizartinib, sorafenib, and sunitinib inhibitors are displayed in Figures S8–S10, respectively. The quizartinib inhibitor shows better interaction with mutants D835H and D835V compared to native and other FLT3 mutants (Table S7). Except for these two mutations (D835H and D835V), all other mutations might be lost the interaction and become resistant to quizartinib inhibitor (Table S7). The mutants D835G, D835I, D835V, and D835Y show better interaction with sorafenib inhibitor than native and other FLT3 proteins (Table S8), whereas the other mutants (D835A, D835E, D835F, D835H, D835V, and D835Y) might lose the interaction with sorafenib inhibitor and could become resistant to it (Table S8). The mutant D835N shows similar interactions as the native FLT3 protein with the sunitinib inhibitor (Table S9). D835E, D835G, D835I, and D835Y show better interaction with sunitinib inhibitor compared to the native and other FLT3 mutant proteins (Table S9). The other FLT3 mutants (D835A, D835F, D835H, and D835V) show lower interaction and a smaller number of H-bonds with the sunitinib inhibitor. Hence, the interaction was lost and could become resistant towards the sunitinib inhibitor (Table S9). In summary, our MD simulations and docking analysis provide details of the structural consequence and their interaction with AML inhibitors which are useful for further development of novel inhibitors to overcome the drug resistance conferred by the D835A/E/F/G/H/I/N/V/Y, FLT3 mutations.

## 4. Methods

### 4.1. Datasets

The protein sequence of FLT3 (ID: P36888) was obtained in FASTA format from the UNIPROT database [59]. A total of 9 mutations, namely, D835A, D835E, D835F, D835G, D835H, D835I, D835N, D835V, and D835Y was retrieved from UNIPROT [59] and COSMIC databases [60]. The crystal structure of FLT3 protein (PDB ID: 1RJB with the resolution of 2.10 Å [61] was obtained from the protein data bank (PDB) [62]. The structure of AML inhibitors [Crenolanib (CID: 10366136); FF-10101 (CID: 90052320); Gilteritinib (CID: 49803313); KW-2449 (CID: 11427553); PLX3397 (CID: 25151352); Ponatinib (CID: 24826799); Quizartinib (CID: 24889392), Sorafenib (CID: 216239); Sunitinib (CID: 5329102); Tandutinib (CID: 3038522)] were downloaded from PubChem database [63] in the 3D SDF format.

### 4.2. Re-Modeling of FLT3 Protein

The available PDB structures of the cytoplasmic domain (amino acid position from 564 to 958) of FLT3 protein consist of many missing residues in the three-dimensional (3D) structures. It is a must to fix the missing residues in the 3D structure of the FLT3 protein to observe the mutational effect at the atomic level. Hence, we used the I-TASSER Server [64] to rebuild the missing residues of FLT3 protein. The I-TASSER server is used to build the 3D structure of a protein from the amino acid sequence. The PDB ID: 1RJB was used as a template to re-model the FLT3 protein. In I-TASSER, the best-modeled structure was selected based on the C-score. The SWISS-PDB viewer tool was used to build the mutant (D835A, D835E, D835F, D835G, D835H, D835I, D835N, D835V, and D835Y) protein structures. Furthermore, the PROCHECK [65] and PROSA [66] tools were used to assess the predicted modeled structures of native and mutant FLT3 protein.

### 4.3. MD Simulation

The GROMACS package [67] was used to conduct an MDS. For the MDS experiments, we used the default parameters that we used in our previous studies [46,47,48,49,50]. The native and mutant FLT3 proteins (D835A, D835E, D835F, D835G, D835H, D835I, D835N, D835V, and D835Y) structures were used as a starting point for MDS. The CHARMM 27 force field was used for the simulation. For the period of computation, the h-atoms were overlooked in the native and mutants FLT3 proteins and solvated in a cubical box with TIP3P water molecules. The native and mutant FLT3 proteins were placed at a margin of 10 Å from the boundaries. Then the native and mutant FLT3 proteins were neutralized by using the genion tool. Furthermore, the energy minimization was performed using the steepest descent algorithm [68] to get a stable protein confirmation. Electrostatic interactions were calculated by the particle mesh Ewald method [69]. The temperature inside the box was controlled using the Berendsen coupling method [70]. Then, the NVT (1000 ps) and NPT (1000 ps) equilibration procedures were performed separately. The water molecules and non-water bonds were regulated using the Parrinello-Rahman barostat pressure coupling method [71] and LINCS [72] algorithms during the equilibration. Lastly, the simulation was conducted out for 200 nanoseconds. To inspect the structural and functional behavior of FLT3 protein upon mutations, we examined the RMSD, RMSF, Rg, SASA, and NH-bonds analysis. We further supported our MD results by carrying out density plot and DCCM matrix and secondary structural analysis to compute the comparative analysis of structural changes of the native and mutant (D835A, D835E, D835F, D835G, D835H, D835I, D835N, D835V, and D835Y) FLT3 proteins. All the MD simulation graphs were generated using the XMGRACE [73] tool. The native and mutants FLT3 protein images at 0 and 200 ns timescale were rendered using PyMol software [74].

### 4.4. Molecular Docking Using AutoDock Vina 4.2

#### 4.4.1. Preparation of Native and Mutant FLT3 Proteins and Inhibitors for Docking

AutoDock tool [75] was used to perform the molecular docking to understand how the mutation at the 835th position interacts with type-I and type-II AML inhibitors. The polar hydrogens, added in the native and mutant FLT3, ahead of docking. We allocated the partial atomic charges to the native and mutant FLT3 proteins using AutoDock Vina 4.2 [76]. The non-polar and polar hydrogen atoms were fused. We applied Gasteiger charges to 10 selected AML inhibitors. The non-polar hydrogens joined and calculated rotatable bonds based on the drug molecule’s nature. The change in free energy (δG) caused by the loss of a torsional degree of freedom upon binding was calculated using TORSDOF. We constructed the peptide backbone bonds in the native and mutant FLT3 proteins. It formed rotatable bonds between selected atoms and all active bonds. The grid maps endorsed inhibitor (ligand) binding and spacing was adjusted to 0.9. We increased the grid size to 31 × 28 × 25 points. For docking, AutoDock Vina employs interaction maps. Before the docking run, Auto Grid calculated these maps. We measured the interaction energy between each ligand atom and the receptor for the entire binding site, which was discretized using a grid, for each ligand atom type. A probe was positioned at each grid point, and it embedded the protein in a 3D grid. At each grid point, the protein’s interaction energy was allocated, and the affinity for each ligand was determined.

#### 4.4.2. Docking of Native and Mutant FLT3 Proteins with AML Inhibitors

Automated docking software AutoDock Vina [76] 4.2 was used to evaluate the binding affinity of 10 AML inhibitors (Crenolanib, FF-10101, Gilteritinib, KW-2449, PLX3397, Ponatinib, Quizartinib, Sorafenib, Sunitinib, and Tandutinib) with native and mutant (D835A, D835E, D835F, D835G, D835H, D835I, D835N, D835V, and D835Y) FLT3 proteins. Using empirical-free energy functions and the Lamarckian genetic algorithm, the docking energy of all inhibitors (ligand molecules) was calculated. Based on different electrostatic, Vander Waal, hydrogen bonding, and desolvation effects, these tools measure the binding-free energy (δG). For each docking run, the docking precision was set to “normal precision” and the ligand-docking mode was set to “flexible.” The AutoDock energy measurements were used to assess the stability of each docked pose. Pymol [74] uses to visualize the interaction residues of native and mutant FLT3 proteins with AML inhibitors.

## 5. Conclusions

In this study, we investigated how mutations on the 835th residue of FLT3 proteins undergo structural transition and affect the interaction with AML inhibitors. As a result of MD simulation, FLT3 protein loses its stability and becomes more flexible. Furthermore, the docking results confirm that a mutation on the 835th residue of FLT3 proteins influences the interaction and shows relapse and resistance responses with AML inhibitors. Certain mutations show increased interaction and relapse response with certain AML inhibitors, and several other mutations show decreased interaction and resistance response to certain AML inhibitors. The collective findings in this study offer perceptions into the conformational and functional changes induced by mutations at the 835th position of FLT3 proteins. Correspondingly, the study also provides a better understanding of the mutational mechanisms of different D835 mutations on FLT3 proteins, which induces AML and can be used to develop a personalized therapeutic strategy to treat AML patients.

## Figures and Tables

**Table 1:** Average values of RMSD, RMSF, Rg, SASA, Density and number of hydrogen bonds (NH-bonds), of Native and mutants FLT3.

Type of Protein	Parameters					
	RMSD (nm)	RMSF	RG (nm)	SASA	Density	NH-Bond
Native	0.32 ± 0.02	0.12 ± 0.09	2.15 ± 0.02	210.25 ± 5.50	38.8	284.85 ± 9.20
D835A	0.34 ± 0.07	0.16 ± 0.15	2.18 ± 0.01	219.51 ± 3.77	26.5	279.91 ± 9.17
D835E	0.35 ± 0.04	0.17 ± 0.14	2.18 ± 0.02	217.02 ± 5.13	21.8	276.11 ± 9.09
D835F	0.39 ± 0.04	0.16 ± 0.12	2.19 ± 0.01	217.02 ± 4.94	23.2	276.58 ± 8.99
D835G	0.47 ± 0.07	0.17 ± 0.13	2.19 ± 0.02	216.16 ± 5.28	31.6	283.47 ± 9.95
D835H	0.40 ± 0.04	0.16 ± 0.16	2.20 ± 0.02	215.94 ± 6.51	33.8	281.06 ± 11.23
D835I	0.45 ± 0.17	0.22 ± 0.22	2.21 ± 0.03	218.12 ± 5.48	25	275.78 ± 9.15
D835N	0.41 ± 0.05	0.15 ± 0.11	2.18 ± 0.04	214.24 ± 3.98	31.9	283.65 ± 8.96
D835V	0.41 ± 0.09	0.19 ±0.17	2.19 ± 0.01	222.08 ± 5.96	21.3	277.54 ± 9.04
D835Y	0.35 ± 0.03	0.13 ± 0.09	2.17 ± 0.01	215.21 ± 4.66	35.2	279.04 ± 8.85

**Table 2:** The percentage of secondary structural elements of native and mutants FLT3.

Protein Type	Secondary Structures of Protein			
	Helix	Beta-Sheets	Coil	Turn
Native	155 (39%)	74 (18%)	168 (42%)	80 (2%)
D835A	139 (35%)	60 (15%)	196 (49%)	104 (26%)
D835E	145 (36%)	68 (17%)	182 (46%)	124 (31%)
D835F	152 (38%)	74 (18%)	169 (46%)	88 (22%)
D835G	147 (37%)	66 (16%)	182 (46%)	120 (30%)
D835H	153 (38%)	69 (17%)	173 (44%)	96 (24%)
D835I	141 (35%)	67 (16%)	187 (47%)	88 (22%)
D835N	152 (38%)	63 (15%)	176 (44%)	96 (24%)
D835V	150 (37%)	68 (17%)	177 (44%)	88 (22%)
D835Y	150 (37%)	74 (18%)	175 (44%)	100 (25%)

**Table 3:** Binding energy of native and mutant FLT3 proteins (200 ns) with inhibitors.

Protein Type (0 ns)	Inhibitor’s Name									
	Crenolanib	FF-10101	Gilteritinib	KW-2449	PLX3397	Ponatinib	Quizartinib	Sorafenib	Sunitinib	Tandutinib
Native	−8.9	−8.3	−8.5	−9.9	−9.7	−10.3	−9.5	−10.4	−8.3	−9.6
D835A	−8.5	−5.8	−6.7	−8.8	−9.6	−7.5	−7.3	−8.3	−6.4	−7.1
D835E	−10.4	−7.7	−7.3	−10.7	−10.1	−7.9	−6.4	−8.3	−9.3	−7.5
D835F	−9.7	−7.8	−8.1	−9.2	−9.2	−8.4	−8.4	−9.2	−8.1	−7.4
D835G	−10.2	−5.9	−7.6	−9.9	−9.9	−8.4	−3.8	−10.5	−9.4	−7.2
D835H	−9.2	−7.6	−9.3	−8.3	−9.0	−10.1	−9.5	−9.5	−8.0	−8.7
D835I	−9.0	−8.6	−8.0	−8.7	−9.9	−10.0	−8.2	−10.8	−8.4	−9.8
D835N	−9.6	−8.2	−4.8	−9.5	−9.8	−7.7	−5.0	−9.7	−8.3	−8.6
D835V	−10.4	−8.8	−9.6	−10.6	−10.1	−12.2	−10.5	−10.8	−9.0	−9.9
D835Y	−8.9	−8.3	−8.9	−9.5	−10.1	−9.9	−11.1	−10.4	−8.7	−9.3

## Supplementary Materials

The following are available online at https://www.mdpi.com/article/10.3390/ijms22147602/s1.
