# Supplementary material for: The Structural Effect of FLT3 Mutations at 835th Position and Their Interaction with Acute Myeloid Leukemia Inhibitors: In Silico Approach
Source: Int J Mol Sci. 2021 Jul 16;22(14):7602. doi: 10.3390/ijms22147602 (PMC8303888; doi:10.3390/ijms22147602)

Table S1: The docking score, number of B-bonds, and interactive residues of native and mutant of FLT3 proteins with Crenolanib inhibitor

Complex Types	Parameters		
	docking score (kcal/mol)	No of H-Bonds	Interactive residues
Native-Crenolanib	-8.9	3	ASN-626, LYS-644, ALA-833
D835A-Crenolanib	-8.5	2	ILE-632, ASN-676
D835E-Crenolanib	-10.4	3	LYS-614, PHE-760, ALA-833
D835F-Crenolanib	-9.7	1	TYR-842
D835G-Crenolanib	-10.2	2	GLN-640, ASN-758
D835H-Crenolanib	-9.2	2	HIS-756, ASP-764
D835I-Crenolanib	-9.0	3	GLU-661, VAL-801, HIS-809
D835N-Crenolanib	-9.6	1	LYS-644
D835V-Crenolanib	-10.4	2	ARG-704, ALA-833
D835Y-Crenolanib	-8.9	2	ARG-807, ASN-887

Complex Types (0ns)	Parameters		
	docking score (kcal/mol)	No of H-Bonds	Interactive residues
Native-FF-10101	-8.3	2	ASN-626, CYS-695
D835A-FF-10101	-5.8	1	SER-670
D835E-FF-10101	-7.7	2	ALA-620, HIS-809
D835F-FF-10101	-7.8	2	CYS-694, GLU-765
D835G-FF-10101	-5.9	1	GLN-640
D835H-FF-10101	-7.6	1	GLU-765
D835I-FF-10101	-8.6	2	GLU-661, VAL-808
D835N-FF-10101	-8.2	0	-
D835V-FF-10101	-8.8	3	LYS-644, ASP-698, ARG-834
D835Y-FF-10101	-8.3	2	CYS-694, ARG-815

Table S2: The docking score, number of B-bonds, and interactive residues of native and mutant of FLT3 proteins with FF-10101inhibitor

Table S3: The docking score, number of B-bonds, and interactive residues of native and mutant of FLT3 proteins with Gilteritinib inhibitor

Complex Types (0ns)	Parameters		
	docking score (kcal/mol)	No of H-Bonds	Interactive residues
Native- Gilteritinib	-8.5	2	GLU-765, ALA-833
D835A-Gilteritinib	-6.7	1	GLU-692
D835E-Gilteritinib	-7.3	2	GLU-654, LEU-832
D835F-Gilteritinib	-8.1	3	CYS-694, ASP-698
D835G-Gilteritinib	-7.6	2	CYS-835, ASP-839
D835H-Gilteritinib	-9.3	2	GLU-765, ARG-815
D835I-Gilteritinib	-8.0	2	PHE-621, GLY-831
D835N-Gilteritinib	-4.8	1	ASP-829
D835V-Gilteritinib	-9.6	3	LEU-616, ASP-698, LEU-755
D835Y-Gilteritinib	-8.9	2	ASP-750, ASN-887

Table S4: The docking score, number of B-bonds, and interactive residues of native and mutant of FLT3 proteins with KW-2449 inhibitor

Complex Types (0ns)	Parameters		
	docking score (kcal/mol)	No of H-Bonds	Interactive residues
Native- KW-2449	-9.9	2	GLU-692, HIS-761
D835A- KW-2449	-8.8	1	GLN-667
D835E- KW-2449	-10.7	2	TYR-693, ASP-829
D835F- KW-2449	-9.2	1	TYR-842
D835G- KW-2449	-9.9	1	CYS-694
D835H- KW-2449	-8.3	1	GLU-765
D835I- KW-2449	-8.7	1	GLU-661
D835N- KW-2449	-9.5	2	GLU-692, SER-736
D835V- KW-2449	-10.6	2	TYR-591, ILE-827
D835Y- KW-2449	-9.5	1	CYS-694

Table S5: The docking score, number of B-bonds, and interactive residues of native and mutant of FLT3 proteins with PLX3397 inhibitor

Complex Types (0ns)	Parameters		
	docking score (kcal/mol)	No of H-Bonds	Interactive residues
Native- PLX3397	-9.7	2	LEU-616, CYS-694
D835A- PLX3397	-9.6	1	SER-806
D835E- PLX3397	-10.1	3	CYS-694, ASP-698, ARG-815
D835F- PLX3397	-9.2	2	CYS-694, HIS-761
D835G- PLX3397	-9.9	2	ASP829, GLY-831
D835H- PLX3397	-9.0	1	ASP-698
D835I- PLX3397	-9.9	1	ILE-827
D835N- PLX3397	-9.8	1	LEU-616
D835V- PLX3397	-10.1	2	CYS-695
D835Y- PLX3397	-10.1	3	LEU-616, ASP-750

Table S6: The docking score, number of B-bonds, and interactive residues of native and mutant of FLT3 proteins with Ponatinib inhibitor

Complex Types (0ns)	Parameters		
	docking score (kcal/mol)	No of H-Bonds	Interactive residues
Native- Ponatinib	-10.3	2	CYS-695, GLU-765
D835A- Ponatinib	-7.5	1	GLY-669
D835E- Ponatinib	-7.9	1	ARG-834
D835F- Ponatinib	-8.4	1	CYS-695
D835G- Ponatinib	-8.4	1	ARG-834
D835H- Ponatinib	-10.1	2	ASP-698, ASN-701
D835I- Ponatinib	-10.0	1	ASP-829
D835N- Ponatinib	-7.7	1	ASP-829
D835V- Ponatinib	-12.2	2	TYR-693, ASP-698
D835Y- Ponatinib	-9.9	1	LEU-616

Table S7: The docking score, number of B-bonds, and interactive residues of native and mutant of FLT3 proteins with Quizartinib inhibitor

Complex Types (0ns)	Parameters		
	docking score (kcal/mol)	No of H-Bonds	Interactive residues
Native-Quizartinib	-9.5	2	GLN-640, ARG-834
D835A-Quizartinib	-7.3	1	LYS-826
D835E-Quizartinib	-6.4	1	SER-653
D835F-Quizartinib	-8.4	1	CYS-695
D835G-Quizartinib	-3.8	0	-
D835H-Quizartinib	-9.5	3	CYS-695, ARG-834, ASN-841
D835I-Quizartinib	-8.2	1	GLU-692
D835N-Quizartinib	-5.0	1	VAL-675
D835V-Quizartinib	-10.5	2	CYS-695, ASP-698
D835Y-Quizartinib	-11.1	1	GLU-692

Table S8: The docking score, number of B-bonds, and interactive residues of native and mutant of FLT3 proteins with Sorafenib inhibitor

Complex Types (0ns)	Parameters		
	docking score (kcal/mol)	No of H-Bonds	Interactive residues
Native-Sorafenib	-10.4	2	CYS-695, GLU-765
D835A-Sorafenib	-8.3	1	GLU-672
D835E-Sorafenib	-8.3	1	TYR-842
D835F-Sorafenib	-9.2	2	ASP-829
D835G-Sorafenib	-10.5	4	SER-653, GLU-661
D835H-Sorafenib	-9.5	1	ASN-701
D835I-Sorafenib	-10.8	3	LEU-616, GLU-661, ASP-829
D835N-Sorafenib	-9.7	2	VAL-808
D835V-Sorafenib	-10.8	2	ASP-698, ARG-815
D835Y-Sorafenib	-10.4	2	LEU-616, ARG-704

Table S9: The docking score, number of B-bonds, and interactive residues of native and mutant of FLT3 proteins with Sunitinib inhibitor.

Complex Types (0ns)	Parameters		
	docking score (kcal/mol)	No of H-Bonds	Interactive residues
Native-Sunitinib	-8.3	2	CYS-694, HIS-761
D835A-Sunitinib	-6.4	1	GLU-692
D835E-Sunitinib	-9.3	3	GLU-654, ASP-811, GLY-831
D835F-Sunitinib	-8.1	2	HIS-761, GLU-765
D835G-Sunitinib	-9.4	2	LEU-616, ARG-815
D835H-Sunitinib	-8.0	1	ARG-815
D835I-Sunitinib	-8.4	2	LYS-644, ASP-829
D835N-Sunitinib	-8.3	2	LYS-644, HIS-809
D835V-Sunitinib	-9.0	1	ASP-829
D835Y-Sunitinib	-8.7	3	ASP-698, ASN-701, ASP-750

Complex Types (0ns)	Parameters		
	docking score (kcal/mol)	No of H-Bonds	Interactive residues
Native-Tandutinib	-9.6	2	GLU-692, CYS-694
D835A-Tandutinib	-7.1	1	LYS-826
D835E-Tandutinib	-7.5	0	-
D835F-Tandutinib	-7.4	1	GLU-776
D835G-Tandutinib	-7.2	1	TYR-693
D835H-Tandutinib	-8.7	1	HIS-756
D835I-Tandutinib	-9.8	1	GLU-661
D835N-Tandutinib	-8.6	1	ASP-829
D835V-Tandutinib	-9.9	2	ASN-758, ARG-834
D835Y-Tandutinib	-9.3	1	CYS-694

Table S10: The docking score, number of B-bonds, and interactive residues of native and mutant of FLT3 proteins with Tandutinib inhibitor

Supplementary Figures

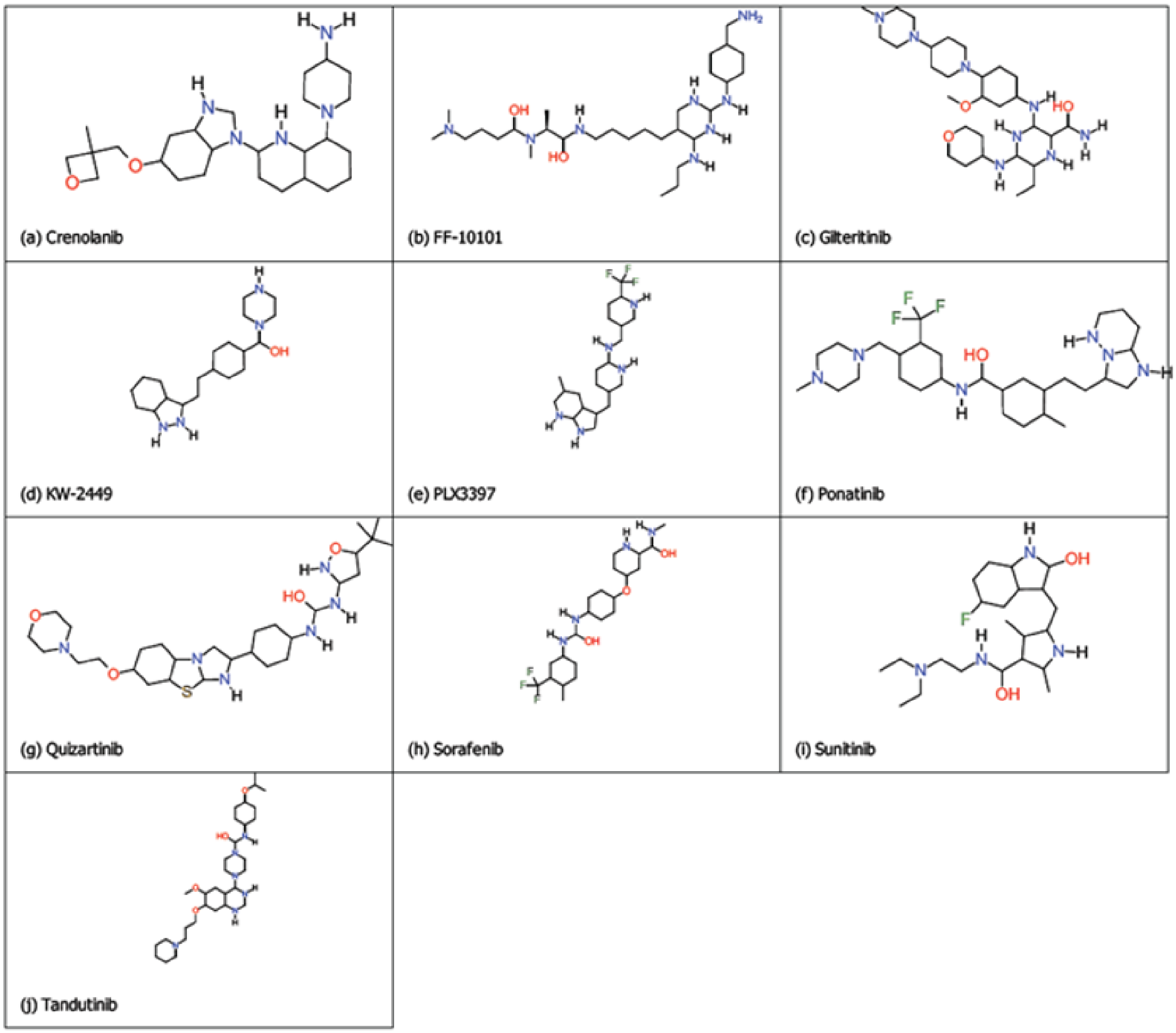

Figure S1: The list of FLT3 inhibitors

Figure S2: The interaction of native FLT3 proteins with AML inhibitors. The native structure was shown in cartoon style (grey) and AML inhibitors were displayed in stick style. The interactive residues of native FLT3 were represented in stick model along with the residues label.

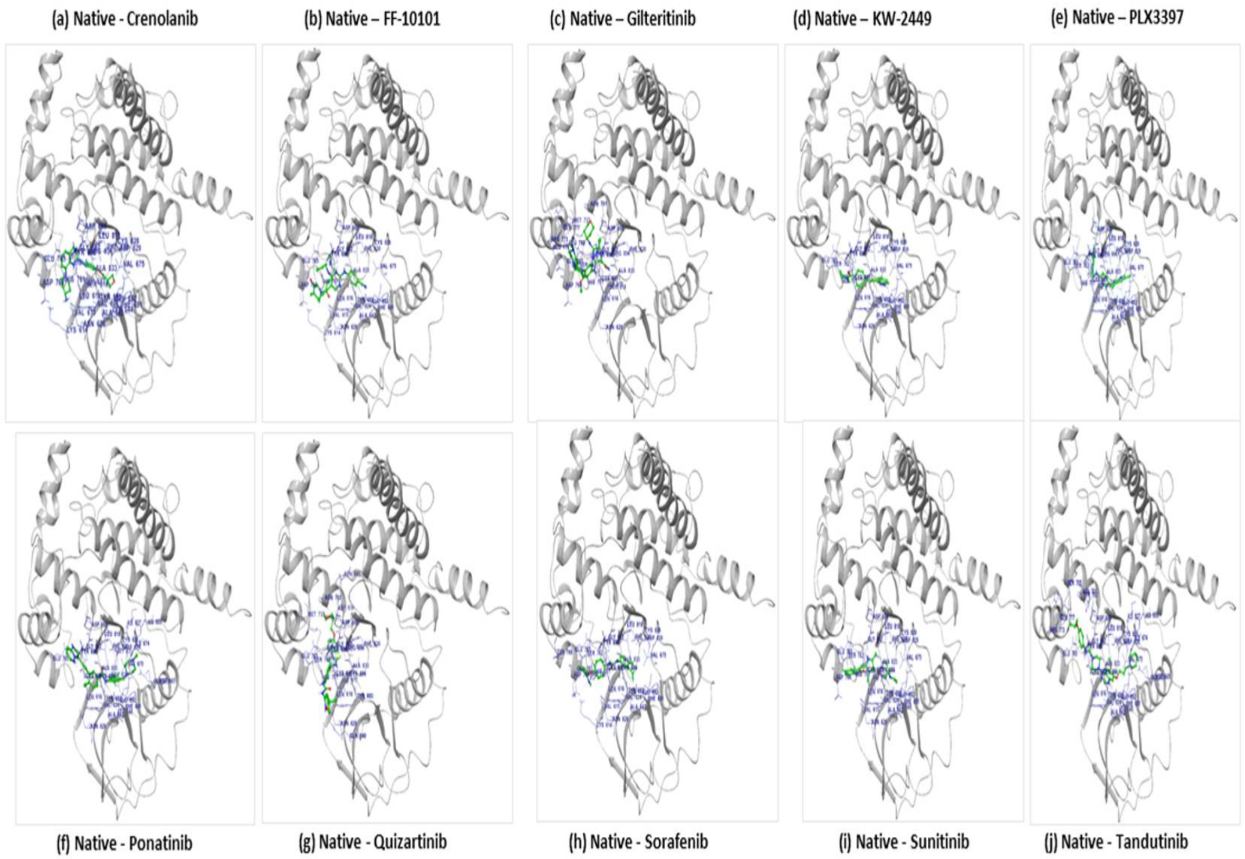

Figure S3: The interaction of mutant (D835A) FLT-3 proteins with AML inhibitors. The D835A-mutant FLT-3 structure was shown in cartoon style (red) and AML inhibitors were displayed in stick style. The interactive residues of native FLT3 were represented in stick model along with the residues label.

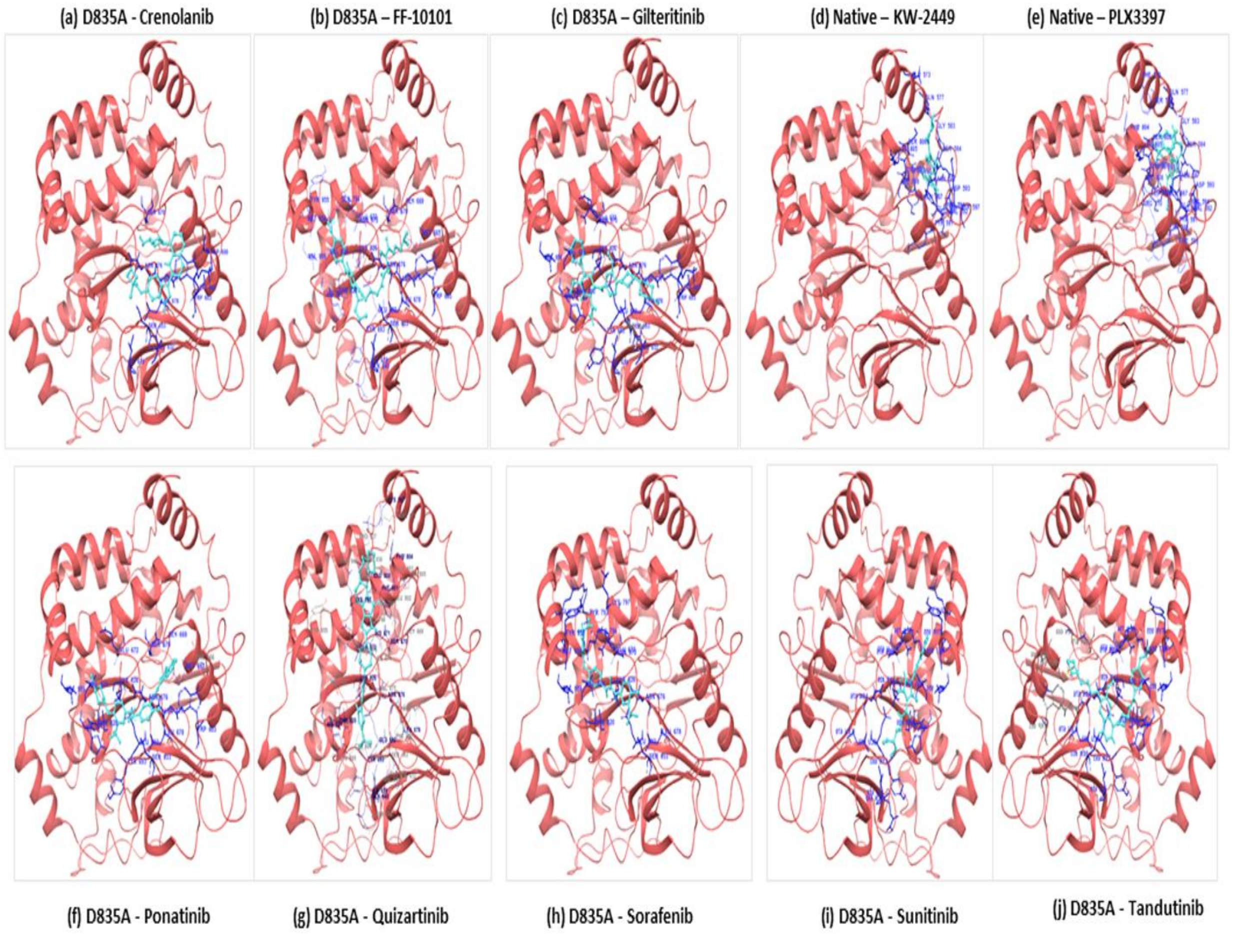

Figure S4: The interaction of 835E-mutant FLT3 proteins with AML inhibitors. The D835E-mutant FLT3 structure was shown in cartoon style (Lemon green) and AML inhibitors were displayed in stick style. The interactive residues of native FLT3 were represented in stick model along with the residues label.

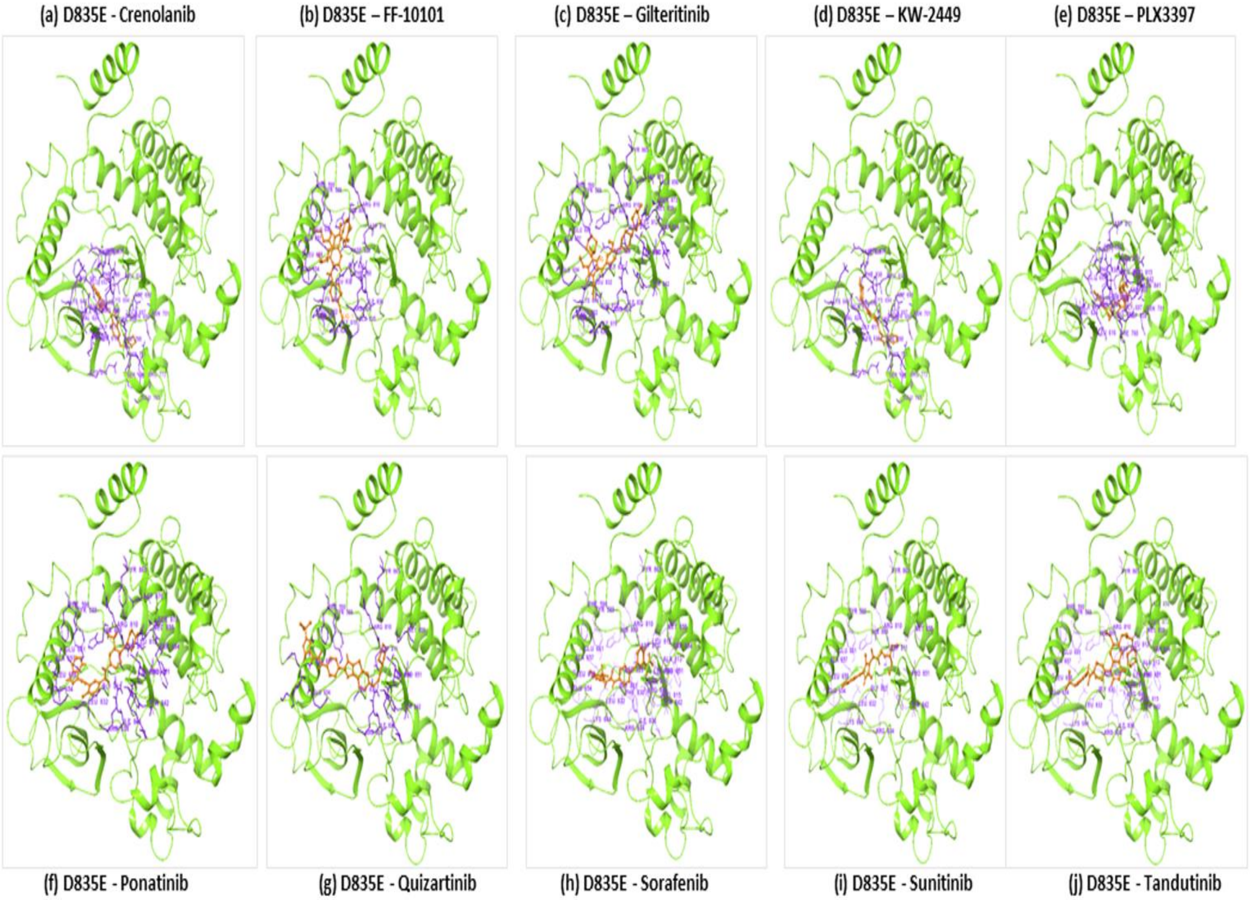

Figure S5: The interaction of D835F-mutant FLT3 proteins with AML inhibitors. The D835F-mutant FLT3 structure was shown in cartoon style (blue) and AML inhibitors were displayed in stick style. The interactive residues of native FLT3 were represented in stick model along with the residues label.

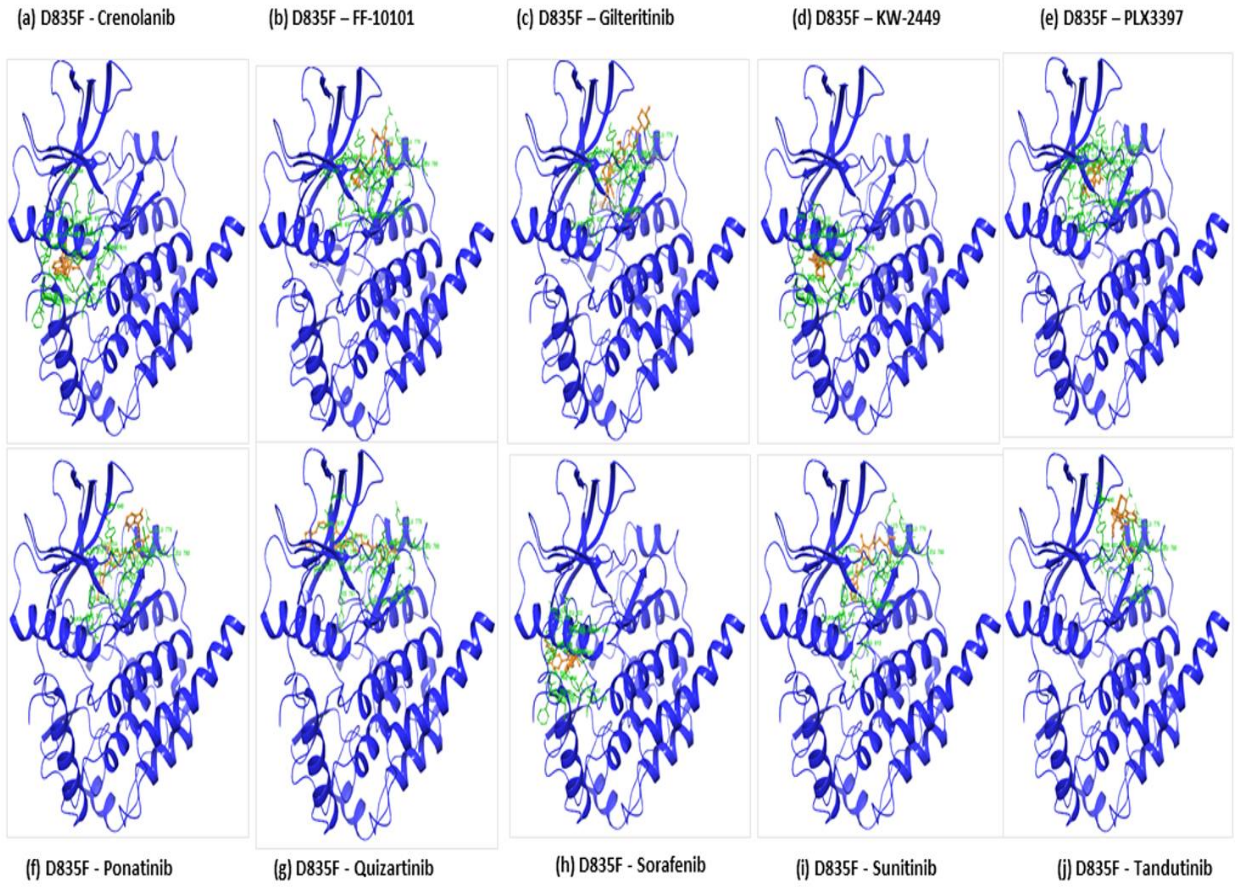

Figure S6: The interaction of D835G-mutant FLT3 proteins with AML inhibitors. The D835G-mutant FLT3 structure was shown in cartoon style (yellow) and AML inhibitors were displayed in stick style. The interactive residues of native FLT3 were represented in stick model along with the residues label.

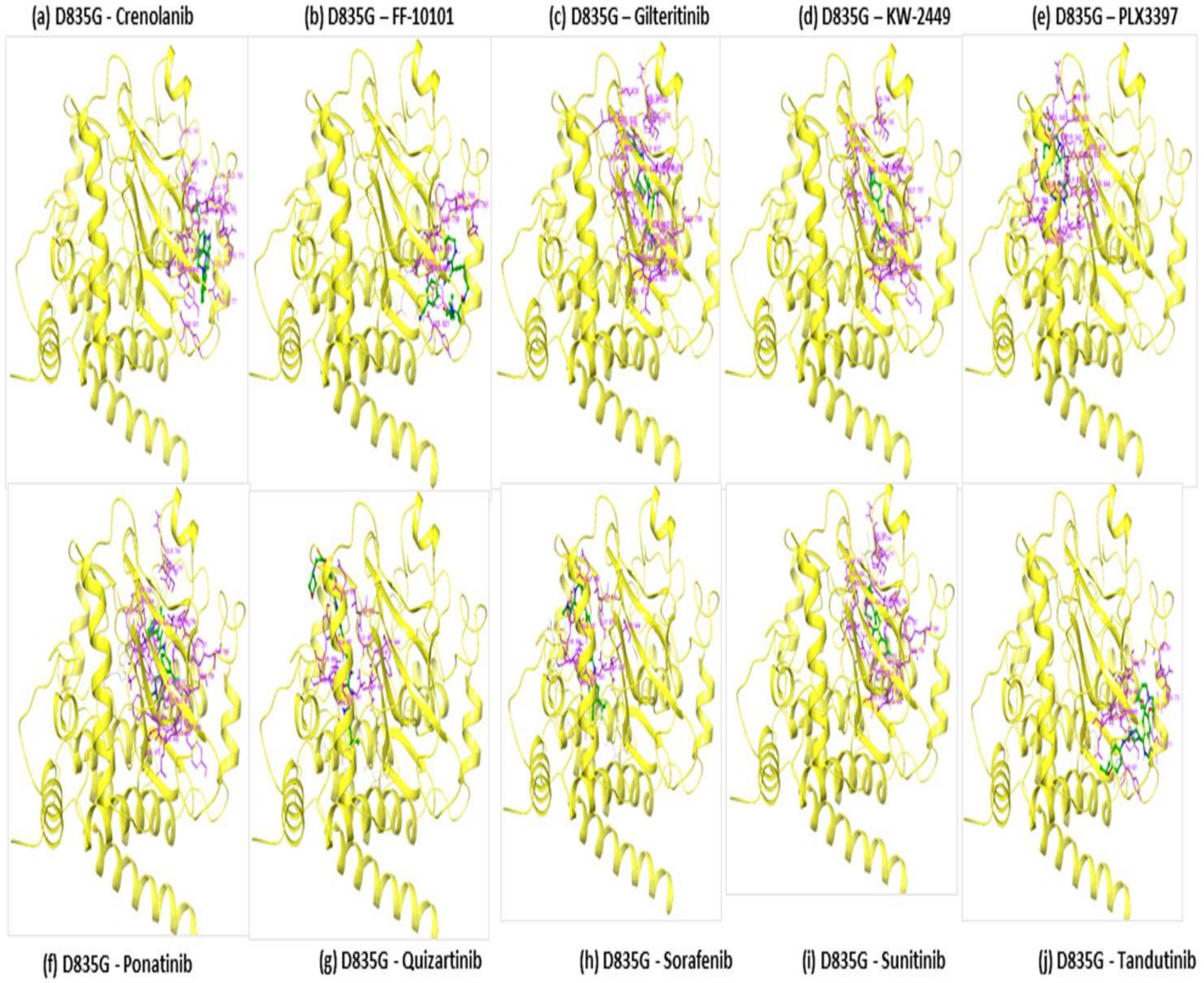

Figure S7: The interaction of D835H-mutant FLT3 proteins with AML inhibitors. The D835H-mutant FLT3 structure was shown in cartoon style (cyan) and AML inhibitors were displayed in stick style. The interactive residues of native FLT3 were represented in stick model along with the residues label.

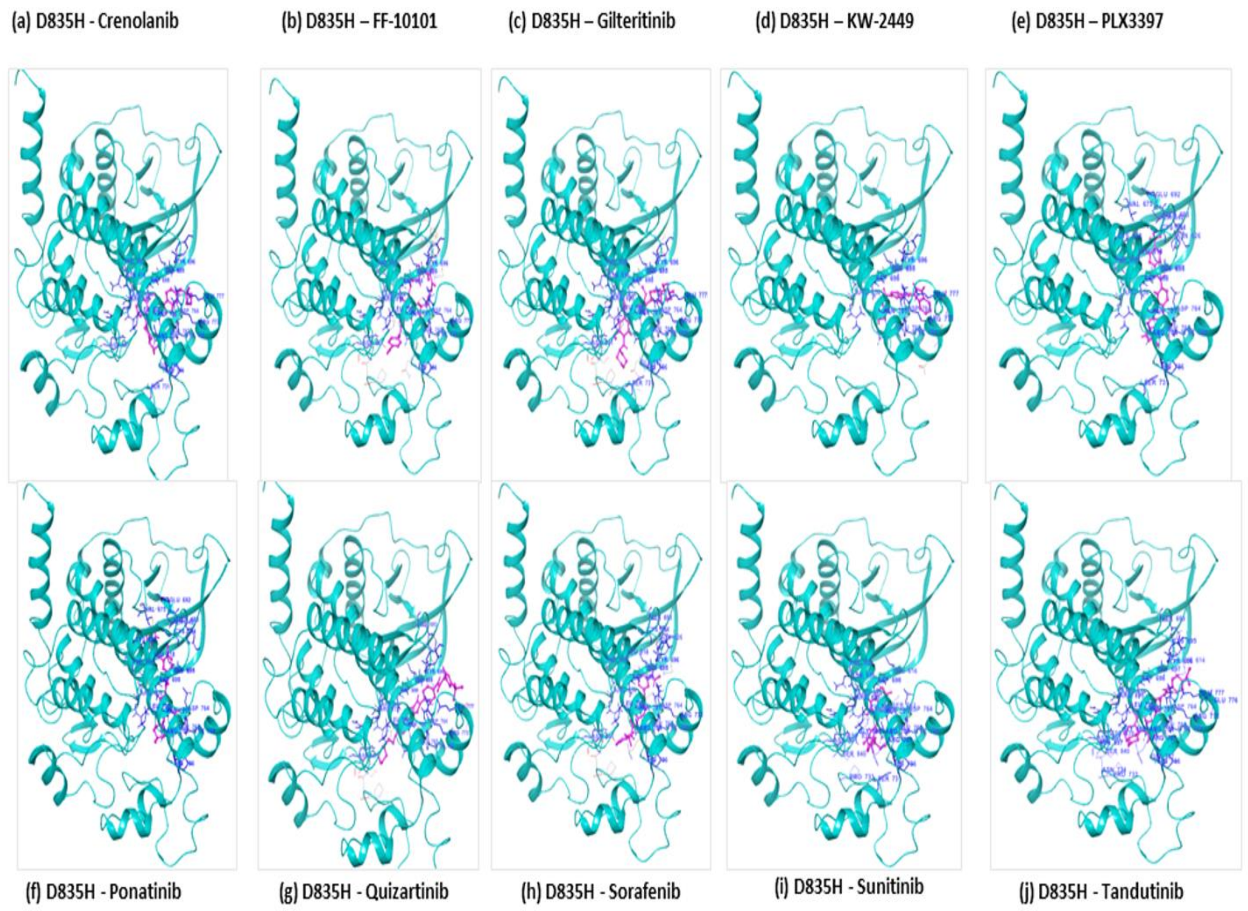

Figure S8: The interaction of D835I-mutant FLT3 proteins with AML inhibitors. The D835I-mutant FLT3 structure was shown in cartoon style (Magenta) and AML inhibitors were displayed in stick style. The interactive residues of native FLT3 were represented in stick model along with the residues label.

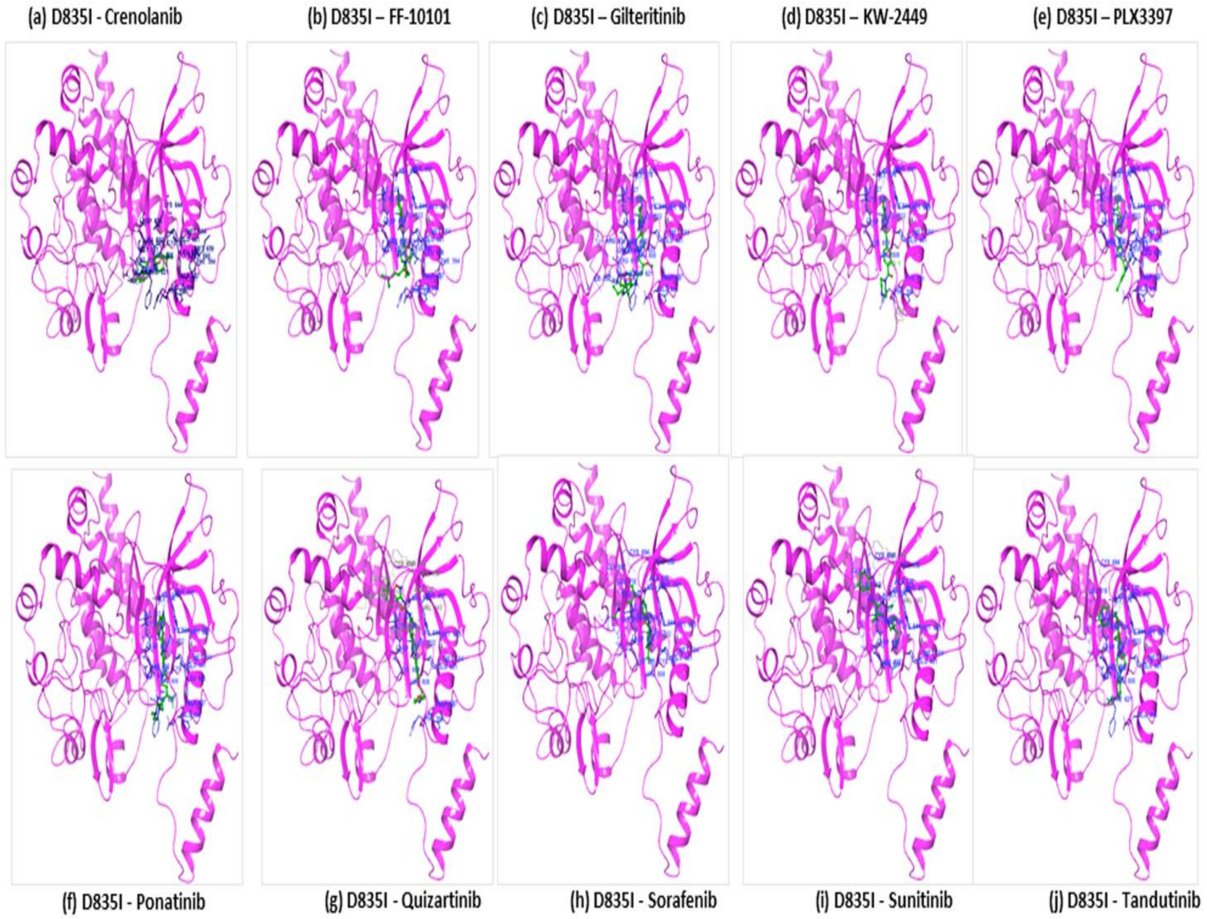

Figure S9: The interaction of D835N-mutant FLT3 proteins with AML inhibitors. The D835N-mutant FLT3 structure was shown in cartoon style (purple) and AML inhibitors were displayed in stick style. The interactive residues of native FLT3 were represented in stick model along with the residues label.

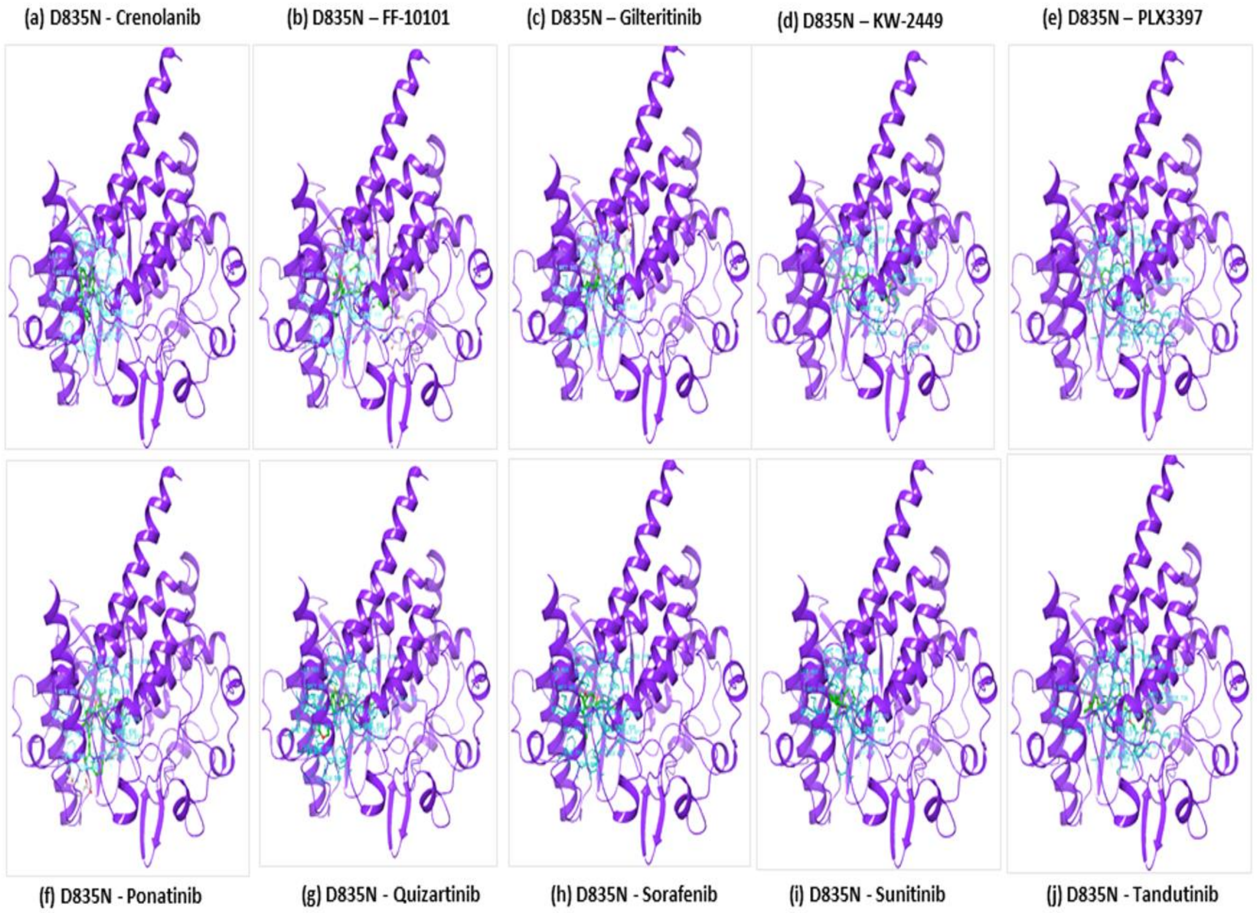

Figure S10: The interaction of D835V-mutant FLT3 proteins with AML inhibitors. The D835V-mutant FLT3 structure was shown in cartoon style (Maroon) and AML inhibitors were displayed in stick style. The interactive residues of native FLT3 were represented in stick model along with the residues label.

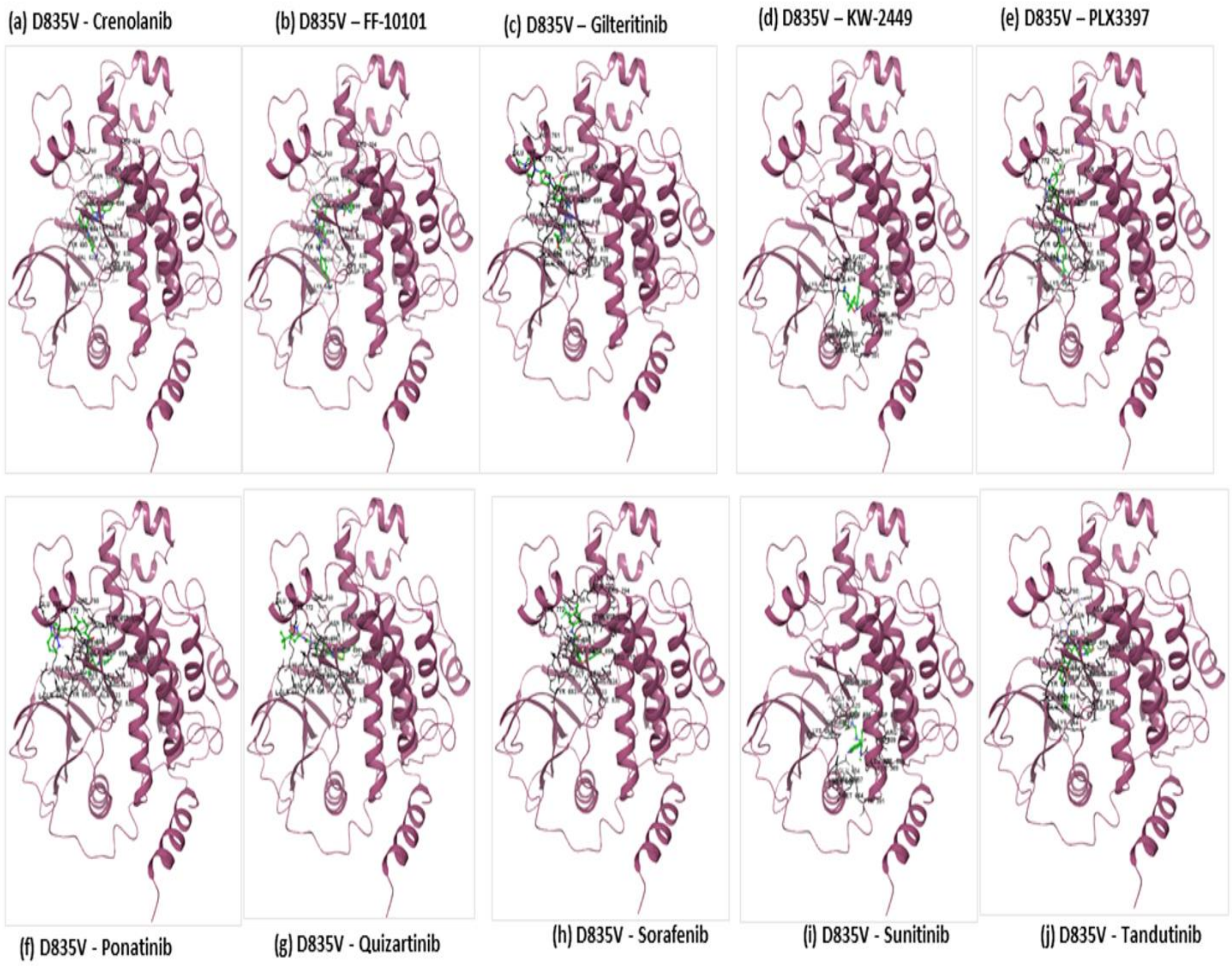

Figure S11: The interaction of D835Y- mutant FLT3 proteins with inhibitors. The D835Y-mutant FLT3 structure was shown in cartoon style (green) and AML inhibitors were displayed in stick style. The interactive residues of native FLT3 were represented in stick model along with the residues label.

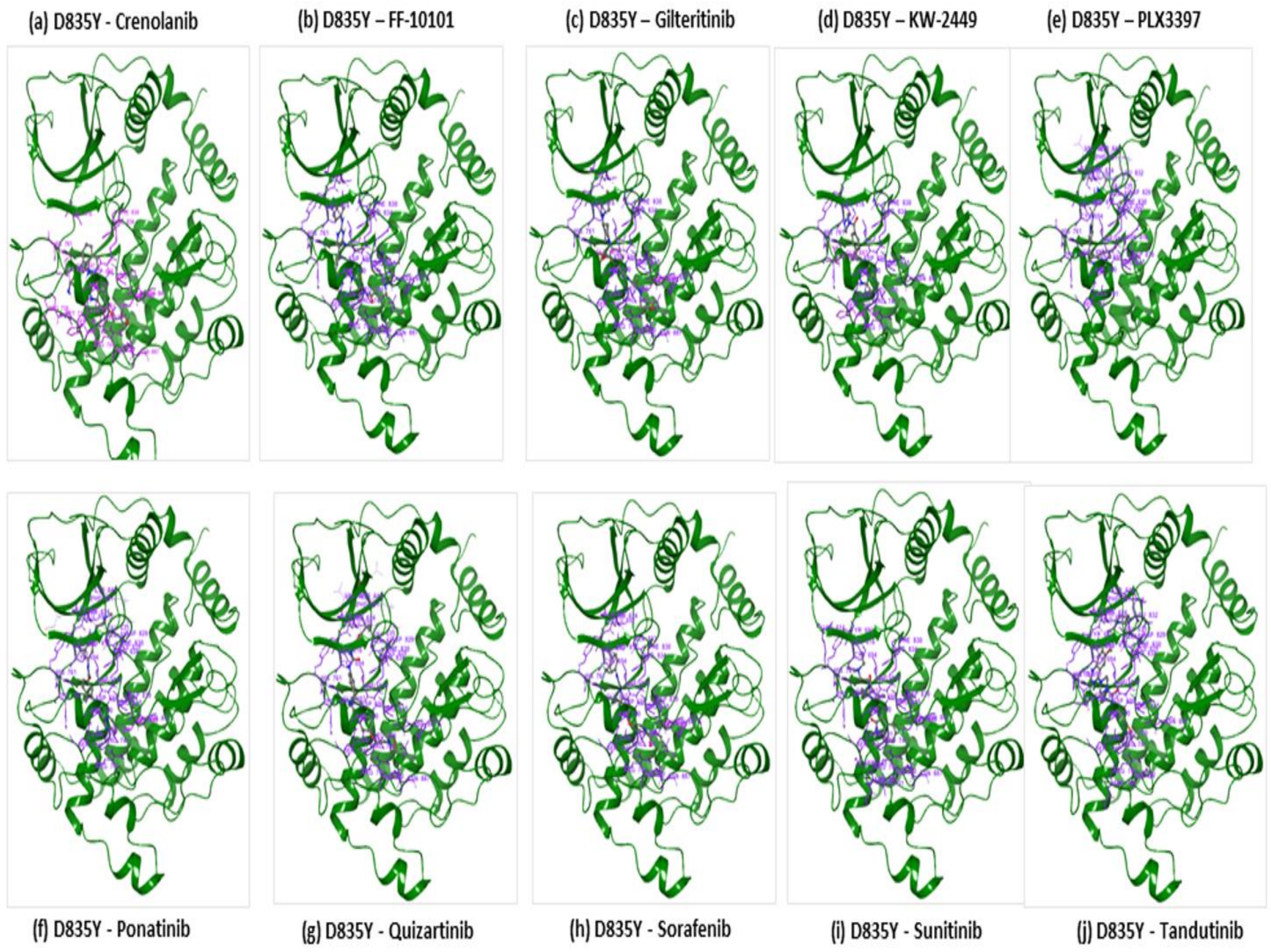

Supplement: Supplementary file 1 [file ijms-22-07602-s001.zip › ijms-1275570-supplementary.pdf]
